# Reduced GATA3 expression associates with immuno‐metabolic alterations and aggressive features in breast cancer

**DOI:** 10.1002/2056-4538.70050

**Published:** 2025-09-29

**Authors:** Anna KM Sæle, Amalie A Svanøe, Cecilie Askeland, Gøril Knutsvik, Lise M Ingebriktsen, Rasmus OC Humlevik, Anette Heie, Turid Aas, Ingeborg Winge, Karin Collett, Ingunn M Stefansson, Erling A Hoivik, Lars A Akslen, Elisabeth Wik

**Affiliations:** ^1^ Centre for Cancer Biomarkers CCBIO, Department of Clinical Medicine, Section for Pathology University of Bergen Bergen Norway; ^2^ Department of Pathology Haukeland University Hospital Bergen Norway; ^3^ Department of Surgery Haukeland University Hospital Bergen Norway

**Keywords:** GATA3, breast cancer, tumor microenvironment, biomarkers

## Abstract

In breast cancer (BC), the transcription factor GATA3 is linked to estrogen receptor (ER) alpha biology, and its loss is associated with aggressive tumor features. Little is reported about potential roles and implications of GATA3 independent of ER, and possible relationships to the BC tumor microenvironment (TME) have not been much explored. Thus, the discovery of novel biomarkers potentially linked to ER and GATA3 functions and predicting aspects of the TME could significantly improve precision in the management of patient subgroups. We examined GATA3 protein and mRNA expression in a large in‐house population‐based BC series (*n* = 837), and in the METABRIC datasets (METABRIC Discovery, *n* = 997 and METABRIC Validation, *n* = 995). Associations with primary BC phenotypes, transcriptional programs, TME features, clinical outcomes, and potentially independent roles of GATA3 are reported. We find that low GATA3 expression associates with aggressive features like increased tumor diameter, higher histological grade, triple negative BC, and a basal‐like (CK5/6 positive) phenotype. Low *GATA3* mRNA expression associated with downregulation of ER‐related genes, upregulation of transcriptional signatures reflecting hypoxia, and enrichment of gene sets reflecting tumor cell proliferation, epithelial‐mesenchymal transition, and stemness. Low GATA3 protein and mRNA expression both associated with overall reduced BC‐specific survival. Notably, low GATA3 expression strongly associated with upregulation of immune checkpoint markers, T‐cell activation, and metabolic alterations not previously described in BC. Gene expression patterns underlying *GATA3*‐low tumors, independent of ER status, reflected activation of immunological and metabolic processes. This study suggests that GATA3 might influence the TME independent of ER status. Our results point to metabolic and immunophenotypic alterations in *GATA3*‐low BCs, in particular with T‐cell activation and increased expression of immune checkpoints. These findings could be relevant for patient selection in the context of immunotherapies and potential targeting of metabolic pathways.

## Introduction

Despite continuous improvements in diagnostic and therapeutic handling of breast cancer (BC), tumors with aggressive features, like large diameter, high histologic grade, triple negative or basal‐like phenotypes, and lymph node metastasis remain a challenge. The tumor microenvironment (TME) holds critical functions in regulating pro‐ and anti‐tumorigenic mechanisms, including immune processes [1, 2, 3, 4, 5]. Several new treatment strategies for aggressive BC are actively being explored, including immune checkpoint inhibition, but the clinical responses are still not satisfactory [6, 7, 8, 9].

GATA3 belongs to the GATA family of transcription factors 1–6 [10] and plays a role in the differentiation of several tissues, including breast [11]. Among ER‐related factors, GATA3 is important in the physiology of breast tissue, luminal epithelial differentiation, and in the pathogenesis of BC [11, 12, 13]. Notably, *GATA3* is one of the most frequently mutated genes in BC, strongly linked to estrogen signaling, and *GATA3* expression correlates with luminal‐like BCs [14, 15, 16, 17]. Previous studies have shown that loss of GATA3 protein and mRNA expression associates with aggressive BC phenotypes [18, 19, 20, 21]. GATA3 is also suggested to induce a growth inhibitory response in triple‐negative breast cancer (TNBC) cell lines [12], and suppress epithelial‐mesenchymal transition (EMT) in BC [22].

Although GATA3 is known to influence immune cell regulation [23, 24] and adipogenesis [11, 25, 26, 27], it is less studied in relation to the complex BC TME. We therefore aimed to explore potentially ER‐independent roles of GATA3 in the TME. By studying protein and mRNA expression in BC cohorts, we found a potential value for GATA3 as a biomarker for improved BC stratification. In particular, our findings suggest novel immuno‐metabolic pathways in certain BC subgroups and point to links between immune responses and metabolic circuits that could potentially be relevant to therapeutic strategies.

## Materials and methods

### Patient series

GATA3 protein or mRNA expression was examined in primary tumors from three cohorts of primary invasive breast carcinoma (supplementary material, Figure S1).

Bergen cohort I is an in‐house population‐based cohort including patients diagnosed with primary BC in the period 1996–2003 in Hordaland County, Norway. Patients diagnosed at age 50–69 years (*n* = 543) were part of the prospective Norwegian mammography screening program in Hordaland County. Detailed descriptions of the series, including clinico‐pathologic variables, have previously been described by our group [28, 29]. The patients below age 50 years at diagnosis (*n* = 355) have recently been described by our group [30]. The immunohistochemical (IHC) markers ER, PR, HER2, and Ki67 were stained and scored as previously described [28, 30]. Complete follow‐up data for cohort I was provided by the Norwegian Cause of Death Registry and is considered to be accurate. The last date of follow‐up was June 30, 2017. All the patients received treatment in accordance with standard protocol at the time, and patients with distant metastasis at diagnosis were excluded. In this cohort, 190 patients (20.8%) died from BC, and 133 patients (14.6%) died due to other causes. Median follow‐up time for survivors was 209.97 months (range 162–257 months).

Ethical approval for the study was granted by the Western Regional Committee of Medical and Health Research Ethics, REK West (REK 2014/1984); informed patient consent was waived. The study was performed in accordance with the guidelines and regulations of the University of Bergen and REK West, and in accordance with the principles of the Declaration of Helsinki.

### Immunohistochemical staining (IHC) for GATA3 protein

GATA3 staining by immunohistochemistry (cohort I; in‐house; *n* = 837/898 cases) was performed using a DAKO autostainer on 4–5 μm tissue microarray (TMA) slides from three cores (diameter 1 mm) of formalin‐fixed paraffin‐embedded (FFPE) primary BC tissue. Whole sections were used for cases with insufficient quality of the TMA sections (*n* = 13). Data were not available for 61 cases due to several factors, including the absence of FFPE blocks, insufficient tumor tissue, and various technical issues encountered during processing. The sections were deparaffinized in xylene, followed by rehydration in alcohols (100%, 96%, and 80%). Epitope retrieval was performed using microwave oven heating (6th Sense) with TRS pH 6 (S 1699) for 20 min. A peroxidase block (S2023) and a protein block (X0909) were applied to limit background staining. The TMAs were incubated at room temperature for 30 min, using a monoclonal mouse antibody against GATA3 (#CM 405 B Biocare Medical, Concord, CA, USA) diluted 1:400 before a secondary antibody EnVision+System‐HRP‐labelled polymer anti‐mouse (K4001) was applied for 30 min. The visualization was performed by diaminobenzidine (DAB, K4009) followed by counterstaining with hematoxylin (S2020).

#### Evaluation of staining

Evaluation of GATA3 nuclear staining was performed by a subjective, semi‐quantitative scoring system based on the intensity of staining (none = 0, weak = 1, moderate = 2, and strong = 3) and the proportion of tumor cell area showing positive staining (<10% = 1, 10–50% = 2, and >50% = 3). A staining index (SI), as described by our group [31, 32], was obtained by multiplying the intensity and area scores (values 0–9). The cut‐off for GATA3 SI high versus low was determined by a frequency distribution test showing bimodal distribution, and in subsequent statistical analysis by evaluating the frequency of case distribution of clinico‐pathologic variables and survival patterns. GATA3 protein expression was considered low for SI <6 and high for SI ≥6. A subset of TMAs (*n* = 51) was scored by two observers (AAS and EW), with good interobserver agreement (kappa‐value 0.72, *p* < 0.001). Normal breast tissue showed heterogeneous GATA3 nuclear staining, observed in lobular and ductal epithelia.

### Gene expression data

Publicly available datasets on global gene expression data from primary invasive BC from the Molecular Taxonomy of Breast Cancer International Consortium, METABRIC (Cohort II: METABRIC Discovery, *n* = 997; Cohort III: METABRIC Validation, *n* = 995) [33], were downloaded and analyzed (Illumina microarray data, log2 transformed) (supplementary material, Figure S1). Multiple probes covering the same gene were collapsed according to the max probe approach [34]. Molecular subtypes identified by PAM50 were available [14, 15], and the normal breast‐like subtype was excluded from analyses. Cut‐off values for analysis of *GATA3*‐high and ‐low mRNA groups were determined after considering the frequency distribution of clinico‐pathologic features and survival patterns of quartiles (METABRIC Discovery: lower quartile, METABRIC Validation: median).

The Kaplan–Meier plotter for BC (www.kmplot.com) [35] was explored for further validation of the prognostic value of *GATA3* mRNA expression [probe; GATA3 (209602_s_at)] – cut‐off median, and relapse‐free survival (RFS) as end point.

#### Gene expression analyses

Significance analysis of microarrays (SAM) [36] was used to explore genes differentially expressed in *GATA3*‐high versus ‐low cases, supplemented by comparative analysis of differentially expressed genes in ER positive and negative cases. Gene set enrichment analysis (GSEA) [34], using the molecular signatures database [MSigDB (https://www.broadinstitute.org/gsea/msigdb) C2 curated, Hallmarks, C5 GO Biological Process ontology, C6 Oncogenic signature gene sets, C7 Immunological signature gene sets, and KEGG C2 curated gene sets], was applied to study gene sets differentially enriched in *GATA3* mRNA‐low tumors. Cytoscape string network analysis [37] was applied for visualization of gene expression interaction data.

The metascape database (http://metascape.org) [38], a gene annotation and analysis resource for identifying functional pathway enrichment, gene expression network components, transcriptional factors' targets, and transcriptional regulators from gene lists, was applied when analyzing gene expression differences in ER‐negative (IHC) and *GATA3* mRNA‐low cases, and differences between GATA3 low luminal A and luminal B molecular subtypes. The express analysis mode was used.

#### Gene expression signatures

Previous published gene expression signatures have been applied: Two hypoxia scores [39, 40] were mapped to the METABRIC data for analyses. An immune cytolytic activity (CYT) score was calculated, defined as the geometric mean of Granzyme A (*GZMA*) and Perforin 1 (*PRF1*) transcriptional expression per sample in the METABRIC Discovery cohort [41].

A gene set reflecting the transcriptional cascade regulating adipogenesis (GSEA, MSigDB/C2/CP/ WP_TRANSCRIPTIONAL_CASCADE_REGULATING_ADIPOGENESIS; METABRIC Discovery cohort) was split into a transcriptional pro‐adipogenesis gene expression sum‐score (eight genes; *EGR2*, *CEBPB*, *CEBPD*, *CREBF1*, *KLF5*, *KLF15*, *CEBPA*, *PPARG*), and an anti‐adipogenesis sum‐score (5 genes; *CEBPG*, *DDIT3*, *GATA2*, *GATA3*, *KLF2*), as categorized by Wiki Pathways (https://www.wikipathways.org/).

#### GATA3‐low signature score

From the list of 150 top ranked genes differentially expressed between *GATA3*‐high and ‐low cases (METABRIC Discovery cohort), we proposed a *GATA3*‐low signature composed of 65 genes; fold change ≥|2.5|; FDR <0.001. The signature score was calculated by subtracting the sum of downregulated genes from the sum of upregulated genes in *GATA*3‐low cases.

#### Connectivity map

Correlations between drug signatures in the Connectivity Map [42, 43, 44] (https://clue.io) and the gene expression pattern in *GATA3*‐low tumors were explored (METABRIC Discovery cohort). Genes differentially expressed (fold change ≥|2.0|; FDR <0.005) between tumor groups of low and high *GATA3* gene expression were included in the Connectivity Map analyses (L1000, MCF7 cell lines).

### Statistical methods

Statistical analyses were performed using SPSS Statistics for Windows, Version 25.0 (IBM Corp., Armonk, NY, USA). Statistical significance was interpreted at the two‐sided *p* value less than 0.05. For categorical variables, associations were evaluated by Pearson's chi‐square test. Odds ratios (OR) and 95% confidence intervals (CI) were estimated. Spearman's rank correlation test was used when comparing bivariate continuous variables, and Spearman's correlation coefficients (*ρ*) were reported. Distribution of continuous variables was compared across categorical variables, using the Mann–Whitney *U* test and Kruskal–Wallis *H* test, and presented by error bars with 95% CI of the mean. Kappa coefficients are presented for interobserver agreement in a subset of patient cohort I (GATA3 IHC assessed cases). In survival analyses, the endpoint was BC‐specific survival in cohorts I–III and recurrence free survival (RFS) in KM‐plotter analysis. Univariate survival analyses were performed applying the Kaplan–Meier method, with significance estimated by the log‐rank test. Multivariate BC‐specific survival analyses were performed using Cox's proportional hazards regression model (‘enter method’). Prognostic variables were included after evaluating their log‐minus‐log plot.

## Results

### Low GATA3 expression associates with aggressive tumor features

**Figure 1:**
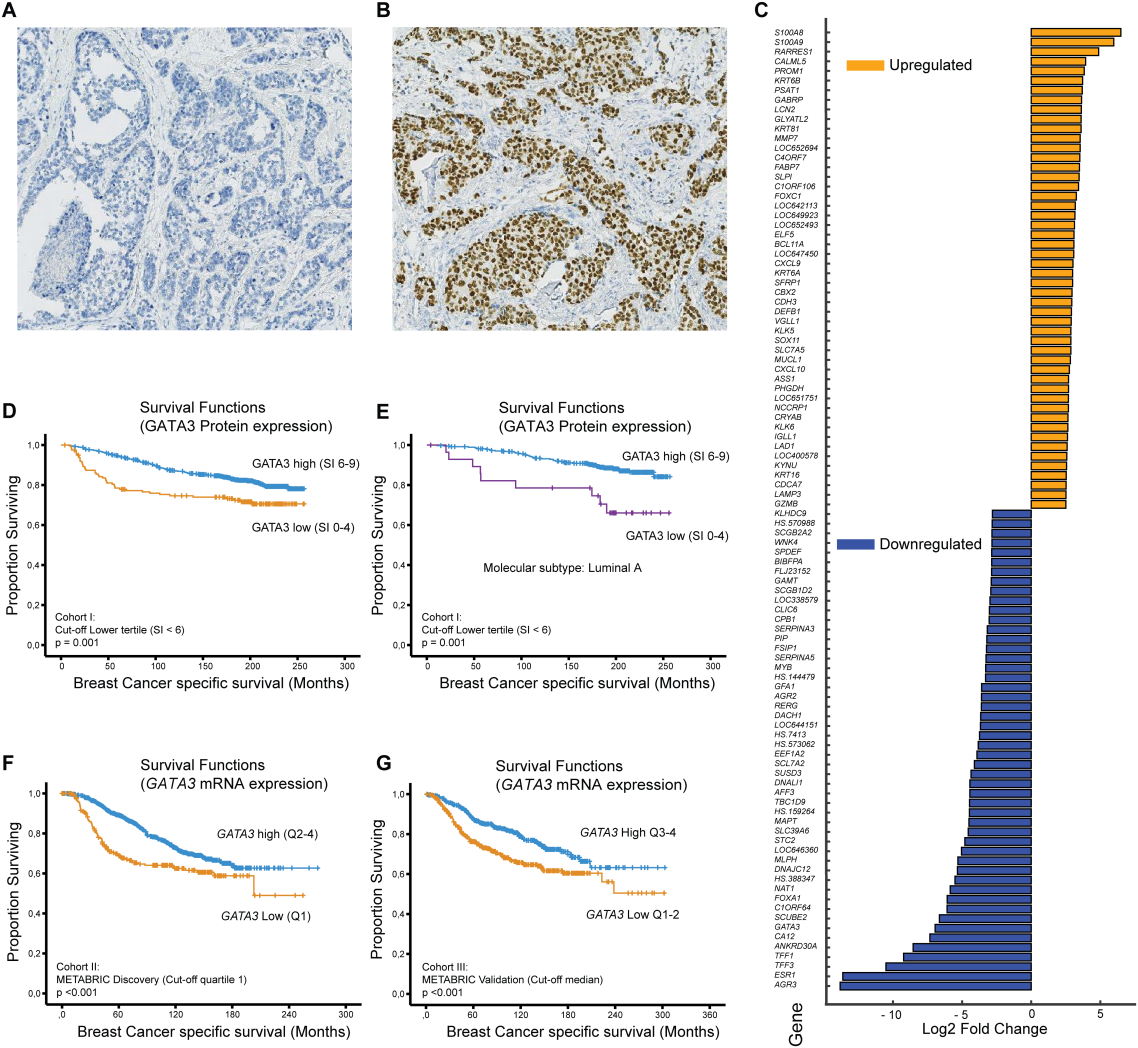
GATA3 protein expression, differentially expressed genes in *GATA3*‐low tumors, and breast cancer specific survival according to GATA3 expression. GATA3 protein expression by immunohistochemistry (Bergen cohort I): (A) negative staining; staining index (SI) 0, and (B) strong nuclear staining; SI 9 in breast cancer cells. (C) Diverging bar plot of the top 50 up‐ and downregulated genes in *GATA3*‐low tumors [METABRIC Discovery cohort (significance analysis of microarray [SAM]); *GATA3* mRNA cutpoint: lower quartile; fold change ≥2; FDR <0.001]. (D, E) Kaplan–Meier univariate breast cancer specific survival according to GATA3 protein expression; overall survival (D) and survival in the luminal A molecular subtype (E); Bergen cohort I, cut‐off SI <6. (F, G) Univariate breast cancer specific survival analysis in cohort II (METABRIC Discovery) according to *GATA3* mRNA expression, cut‐off: lower quartile (F), and cohort III (METABRIC Validation), cut‐off: median (G). *p* values correspond to the log‐rank test.

Immunohistochemical GATA3 protein staining was mainly nuclear and interpreted as low (score index <6) in 18.0% of cases (*n* = 164/837; Bergen cohort I; Figure 1A, B). Low GATA3 protein expression was strongly associated with tumor diameter >20 mm, high histologic grade, increased tumor cell proliferation (by Ki67), ER and PR negativity, and HER2 positivity (*p* ≤ 0.008; Table 1). In contrast, GATA3 protein expression did not show a significant association with lymph node metastases. Notably, low GATA3 protein expression was associated with the triple negative and basal‐like (CK5/6 positive) phenotypes (*p* < 0.001; Table 1).

**Table 1:** Bergen cohort I (in‐house): associations between GATA3 protein expression (IHC) and clinico‐pathologic features as well as molecular markers in breast cancer (*n* = 837)

Variables	GATA3 high (SI 6–9)	GATA3 low (SI 0–4)	OR	(95% CI)	*p* ^†^
	*n* (%)	*n* (%)			
Age					
≥40 years	631 (83.0)	129 (17.0)	1		
<40 years	45 (58.4)	32 (41.6)	3.01	(1.85–4.91)	<0.001
Histologic grade^‡^					
Grades 1 and 2	568 (88.6)	73 (11.4)	1		
Grade 3	100 (54.9)	82 (45.1)	6.38	(4.36–9.33)	<0.001
Tumor diameter^‡^					
≤20 mm	487 (84.8)	87 (15.2)	1		
>20 mm	186 (71.8)	73 (28.2)	2.20	(1.54–3.13)	<0.001
Nodal status^‡^					
Negative	449 (82.1)	98 (17.9)	1		
Positive	221 (78.1)	62 (21.9)	1.28	(0.90–1.84)	NS (0.194)
ER					
Positive (≥10%)	609 (93.1)	45 (6.9)	1		
Negative (<10%)	67 (36.6)	116 (63.4)	23.43	(15.29–35.90)	<0.001
PR					
Positive (≥10%)	541 (92.8)	42 (7.2)	1		
Negative (<10%)	135 (53.1)	119 (46.9)	11.35	(7.61–16.92)	<0.001
HER2^‡^					
Negative	583 (82.2)	126 (17.8)	1		
Positive	83 (70.9)	34 (29.1)	1.90	(1.22–2.95)	0.008
Ki67^‡^					
Low*	405 (88.2)	54 (11.8)	1		
High*	263 (71.1)	107 (28.9)	3.06	(2.12–4.38)	<0.001
Basal‐like (CK5/6)^‡^					
Negative	621 (87.1)	92 (12.9)	1		
Positive	49 (41.9)	68 (58.1)	9.37	(6.1–14.37)	<0.001
Molecular subtypes^‡^					
Lum A	379 (92.9)	29 (7.1)			<0.001
Lum B	191 (92.7)	15 (7.3)			
Lum B HER2+	60 (87.0)	9 (13.0)			
HER2+	23 (47.9)	25 (52.1)			
Triple negative	12 (12.6)	83 (87.4)			

CI, confidence interval; CK5/6: cytokeratin 5/6; ER, estrogen receptor; HER2, human epidermal growth factor receptor 2; *n*, number of patients; OR, odds ratio; PR, progesterone receptor.

* Ki67 count: cut‐off; reference number 30.

^†^ *p* values by Pearson's chi‐squared test.

^‡^ Missing information: Histologic grade: *n* = 14; Tumor diameter: *n* = 4; Nodal status: *n* = 7; HER2: *n* = 11; Ki67: *n* = 8; CK5/6: *n* = 7; Molecular subtypes (IHC): *n* = 11.

Low *GATA3* mRNA levels are also associated with adverse tumor features, such as high histological grade, ER and PR negativity, and HER2 positivity (all *p* < 0.001; METABRIC cohorts; supplementary material, Table S1A, B). Here, low *GATA3* mRNA is associated with lymph node metastasis (*p* ≤ 0.003). Notably, both low GATA3 protein (Bergen cohort I) and mRNA levels (METABRIC cohorts II–III) are significantly associated with the group of young breast cancer patients (below 40 years), with tumors more frequently having low GATA3 compared to breast cancer patients ≥40 years (*p* < 0.001; Table 1; supplementary material, Table S1).

A subgroup of cases with low GATA3 protein expression was ER positive (*n* = 45/161; 28.0%), and a subgroup of ER‐negative tumors showed high GATA3 expression (*n* = 67/676; 9.9%). Low *GATA3* mRNA expression in both ER positive and negative tumors was associated with higher histologic grade (METABRIC Discovery; *p* < 0.001). Combined GATA3‐low (protein)/ER‐negative tumors were associated with increased tumor diameter, higher histologic grade, TNBC, and basal‐like (CK5/6 positive) phenotypes, compared to cases with GATA3 high (protein)/ER‐negative status (all *p* < 0.001, Table 2).

**Table 2:** Bergen cohort I (in‐house): GATA3 expression (IHC) and ER status; associations to basic clinico‐pathological parameters, TNBC, and basal‐like phenotype (*n* = 837)

Variables	ER pos/GATA3 high (SI 6–9)	ER pos/GATA3 low (SI 0–4)	ER neg/GATA3 high (SI 6–9)	ER neg/GATA3 low (SI 0–4)	*p* *
	*n* (%)	*n* (%)	*n* (%)	*n* (%)	
Histological grade^†^					
Grades 1 and 2	530 (88.0)	36 (80.0)	38 (57.6)	37 (33.6)	<0.001
Grade 3	72 (12.0)	9 (20.0)	28 (42.4)	73 (66.4)	
Tumor diameter^†^					
≤20 mm	445 (73.3)	32 (71.1)	42 (63.6)	55 (47.8)	<0.001
>20 mm	161 (26.7)	13 (28.9)	24 (36.4)	60 (52.2)	
Nodal status^†^					
Negative	414 (68.7)	29 (64.4)	35 (52.2)	69 (60.0)	0.024
Positive	189 (31.3)	16 (35.6)	32 (47.8)	46 (40.0)	
Basal like (CK5/6)^†^					
Negative	567 (93.9)	41 (93.2)	54 (81.8)	51 (44.0)	<0.001
Positive	37 (6.1)	3 (6.8)	12 (18.2)	65 (56.0)	
TNBC (ER/PR/HER‐2 negative)					
No	609 (100)	45 (100)	55 (82.1)	33 (28.4)	<0.001
TNBC	0 (0)	0 (0)	12 (17.9)	83 (71.6)	

CK5/6, cytokeratin 5/6; ER, estrogen receptor; *n*, number of patients; TNBC, triple negative breast cancer.

* *p* values by Pearson's chi‐squared test.

^†^ Missing information: Histological grade: *n* = 14; Tumor diameter: *n* = 4; Nodal status: *n* = 7; CK5/6: *n* = 7.

To better understand the strong associations between low *GATA3* expression and aggressive tumor features, we explored the global gene expression data by significance analysis of microarrays (SAM) and GSEA employing METABRIC gene expression data, searching for biological processes related to increased tumor aggressiveness. In the analysis of genes differentially expressed between *GATA3* low versus high cases, ER‐related genes were among the top‐ranked downregulated genes in *GATA3*‐low tumors (METABRIC Discovery; fold change ≤−6.1; FDR <0.001; Figure 1C; supplementary material, Figure S2A–D, Table S2).

We then developed a *GATA3*‐low mRNA signature score consisting of 65 genes differentially expressed between *GATA3*‐low and *GATA3*‐high cases (cutpoint lower quartile; fold change > ±2.5; FDR <0.001; supplementary material, Table S3; see Materials and Methods for score calculation). Gene sets reflecting proliferation, basal‐like features, and characteristics of stemness were enriched in *GATA3*‐low tumors (supplementary material, Tables S4 and S5). Both low *GATA3* gene expression and *GATA3*‐low mRNA signature score strongly associated with the basal‐like molecular subtype (*p* < 0.001; supplementary material, Figure S2E, F).

Hypoxia was significantly enriched in *GATA3*‐low cases (supplementary material, Table S4). Supporting this finding, we demonstrated negative correlations between *GATA3* mRNA expression and high scores of previously published signatures reflecting hypoxia [39, 40] (all cohorts; *p* < 0.001; supplementary material, Figure S2G, H).

### GATA3 expression and patient survival

In all cohorts, low GATA3 protein and *GATA3* mRNA expression associated with overall reduced breast cancer‐specific survival (*p* ≤ 0.001; Figure 1D, F, G). Low *GATA3* also associated with shorter relapse‐free survival (KM‐plotter tool for BC; *p* < 0.001; supplementary material, Figure S3). Of note, low GATA3 protein, but not mRNA expression, associated with reduced BC‐specific survival within the luminal A molecular subtype (*p* = 0.001; Figure 1E). However, by multivariate analysis, including basic clinico‐pathologic variables (histologic grade, tumor diameter, lymph node status) and ER expression, low GATA3 protein or mRNA was not independently associated with reduced disease‐specific survival (data not shown).

### *GATA3* expression in relation to immune response alterations

Several of the top‐ranked up‐regulated genes in *GATA3*‐low tumors were associated with immunological alterations (METABRIC Discovery cohort; Figure 1C; supplementary material, Table S2). By GSEA, gene sets reflecting a mixed pattern, with both activation of immunological responses and programs facilitating an immunosuppressive microenvironment, were enriched in *GATA3*‐low tumors (supplementary material, Table S5).

**Figure 2:**
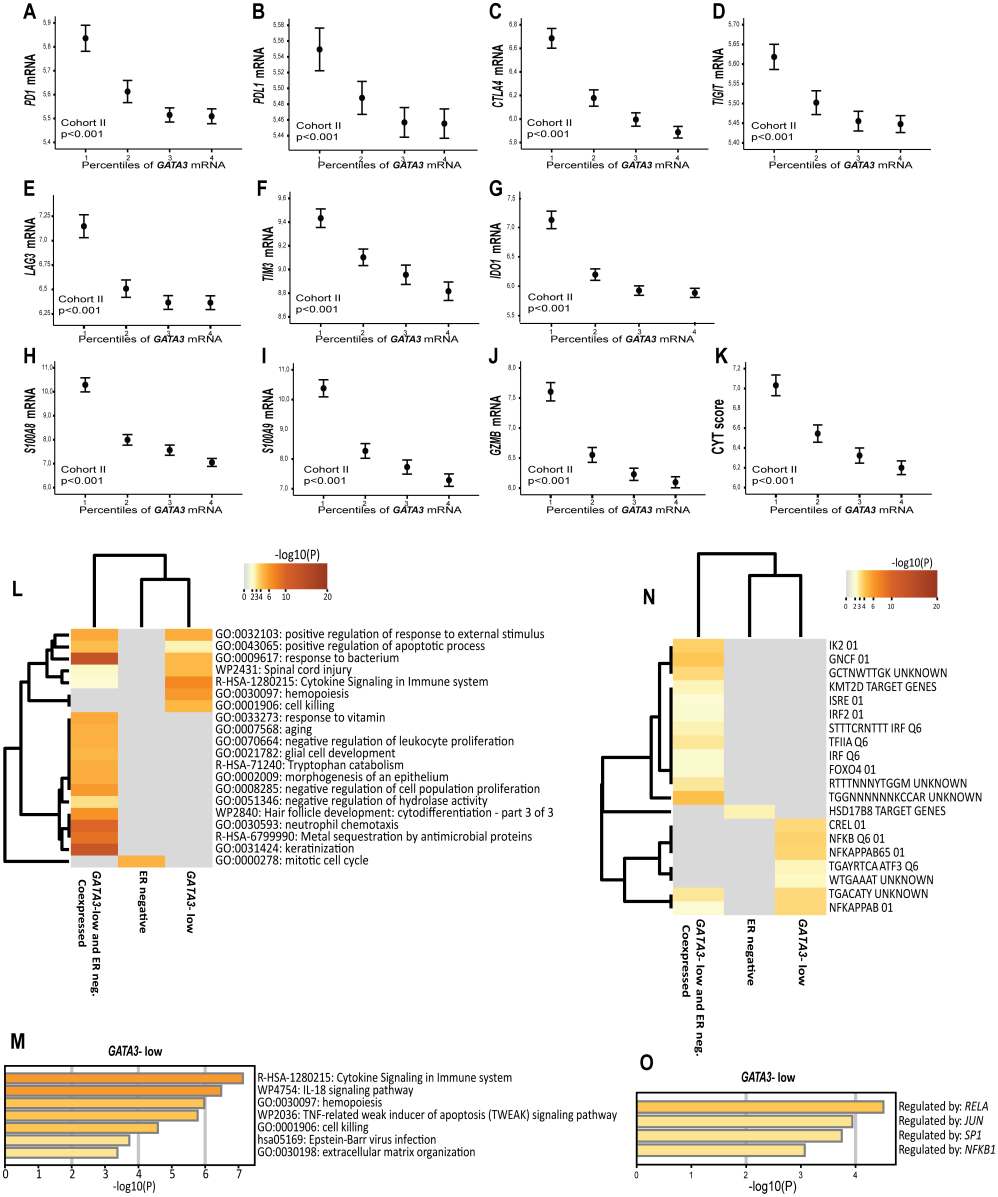
*GATA3* mRNA expression and associations to gene expression of inhibitory immune related transcripts, and cytolytic activity. mRNA expression of (A) *PD1*, (B) *PD‐L1*, (C) *CTLA4*, (D) *TIGIT*, (E) *LAG3*, (F) *TIM3*, (G) *IDO1*, (H) *S100A8*, (I) *S100A9*, (J) *GZMB* and (K) Cytolytic activity (CYT) score, across the expression of *GATA3* mRNA in percentile groups, ranked from percentile group 1 (lowest expression) to percentile group 4 (highest expression). Error‐bars represent 95% confidence interval from the mean, and *p* values by Kruskal–Wallis test. All data from cohort II; METABRIC Discovery (*n*, number of patients = 939, log2 transformed). The normal breast‐like category was excluded. (L–O) Pathway enrichment, enrichment of transcription factor targets, and gene regulators across upregulated differentially expressed genes (DEGs) splitting *GATA3*‐low and ER (IHC) negative tumors (analysis by Metascape); Metabric Discovery; Fold change ≥2; FDR <0.001. (L) Heatmap illustrating the top 20 clusters of functional pathway enrichment across *GATA3*‐low, ER‐negative, and co‐expressed DEGs, and (M) pathway enrichment for upregulated DEGs in *GATA3*‐low cases. (N) Enrichment of transcription factor targets across upregulated DEGs; co‐expressed, and splitting *GATA3*‐low and ER‐negative cases. (O) Enrichment of gene regulators across upregulated DEGs in *GATA*3‐low cases. All heatmaps colored according to the *p* value.

We further asked whether low *GATA3* mRNA expression was related to immune inhibitory transcripts. In METABRIC cohorts, low *GATA3* associated with increased expression of immune checkpoint markers like PD1, CTLA4, TIGIT, PD‐L1, LAG3, TIM3, and IDO1 (all p < 0.001; Figure 2A–G). Low *GATA3* mRNA associated strongly with increased expression of *S100A8/9*, which relates to immunosuppressive function in myeloid‐derived suppressor cells (MDSCs) [45, 46] (Figure 2H, I), and activated signaling pathways as IL6‐JAK‐STAT3, a top‐ranked gene set enriched in *GATA3*‐low tumors (supplementary material, Table S5). Additionally, the T‐cell cytotoxic anti‐tumor effector molecule Granzyme B (GZMB) was among the top‐ranked and up‐regulated genes in *GATA3*‐low tumors (Figure 2J).

We then explored an immune cytolytic activity (CYT) score, defined as the geometric mean of Perforin 1 (*PRF1*) and Granzyme A (GZMA) [41]. Here, low *GATA3* mRNA was strongly associated with increased transcriptional levels of the CYT score, indicating T‐cell activation with increased immune cytolytic activity in *GATA3* low tumors (*p* < 0.001; Figure 2K). A high CYT score was associated with increased expression of transcripts facilitating an immunosuppressive TME (*CTLA‐4*, *PD1*, *PD‐L1*, *TIM3*, *IDO1*, *LAG3*, *TIGIT*, and *S100A8/9*, all *p* < 0.001).

Next, we compared the output of differentially expressed genes (DEGs) between *GATA3* mRNA low and high cases, and between ER positive and negative (IHC) cases. Applying a stringent cut‐off (fold change ≥2.0; FDR < 0.001) revealed 18 genes uniquely upregulated in *GATA3*‐low tumors, not found in ER‐negative cases (supplementary material, Table S6A). The majority of genes were immune‐related and included chemotactic cytokines (*CCL19* and *CCL5*), lymphocyte markers (*CD3D*, *CD79a*), and genes reflecting cytolytic activity (e.g., *GNLY*). Functional pathway analysis confirmed that these samples were enriched in immune‐stimulating pathways (Figure 2L, M). Also, transcription factor targets of the nuclear factor kappa beta (NF‐ĸB) family, including c‐REL, were enriched in the *GATA3*‐low group, and *RELA* was a top‐ranked transcriptional regulator (Figure 2N, O). Widening the fold change (Fch ≥1.5) showed upregulation of immune checkpoint molecules, DEGs reflecting cytolytic activity, and densely connected gene expression networks associated with complement activation, chemokine and interferon signaling in the *GATA3* low group (supplementary material, Table S6B and Figure S4A–F). A fold change ≥2.5 (FDR < 0.001) limited the list of DEGs to six genes only upregulated in *GATA3*‐low tumors, dominated by genes reflecting immunological alterations, like granzyme B (*GZMB*), and the oncometabolite kynureninase (*KYNU*), associated with an inhibitory effect on T‐cells and promotion of Tregs [5] (supplementary material, Table S6C).

### *GATA3* expression in relation to metabolic alterations

Gene sets reflecting activated adipogenesis were enriched in *GATA3*‐low cases, and markers of metabolism, like *FABP7*, *RARRES1*, and *CRABP1* [47, 48, 49], were among the most upregulated genes in *GATA3*‐low cases, as visualized by the strong negative association between these genes and *GATA3* mRNA expression (all *p* < 0.001; supplementary material, Figure 5SA–C). Further, low *GATA3* was strongly associated with the cholesterol synthesis precursor enzyme 3‐hydroxy‐3‐methylglutaryl‐CoA synthase 1 (*HMGCS1*) (*p* < 0.001; supplementary material, Figure S5D), suggested to contribute to dysregulated mevalonate metabolism and stemness in BC models [50].

We then analyzed a gene set reflecting the transcriptional cascade regulating adipogenesis. This gene set was split into a transcriptional pro‐adipogenesis mRNA score (eight genes) and an anti‐adipogenesis score (five genes) (see Materials and Methods). The pro‐adipogenesis score associated with low *GATA3* mRNA expression, and the anti‐adipogenesis score associated with increased expression of *GATA3* mRNA (both *p* < 0.001; supplementary material, Figure S5E, F). In support of this, a high score of genes upregulated in the differentiation of mature adipocytes compared to preadipocytes was significantly associated with low *GATA*3 mRNA expression (*p* < 0.001; supplementary material, Figure S5G).

### Gene expression by *GATA3* and ER status in luminal breast cancer subtypes

**Figure 3:**
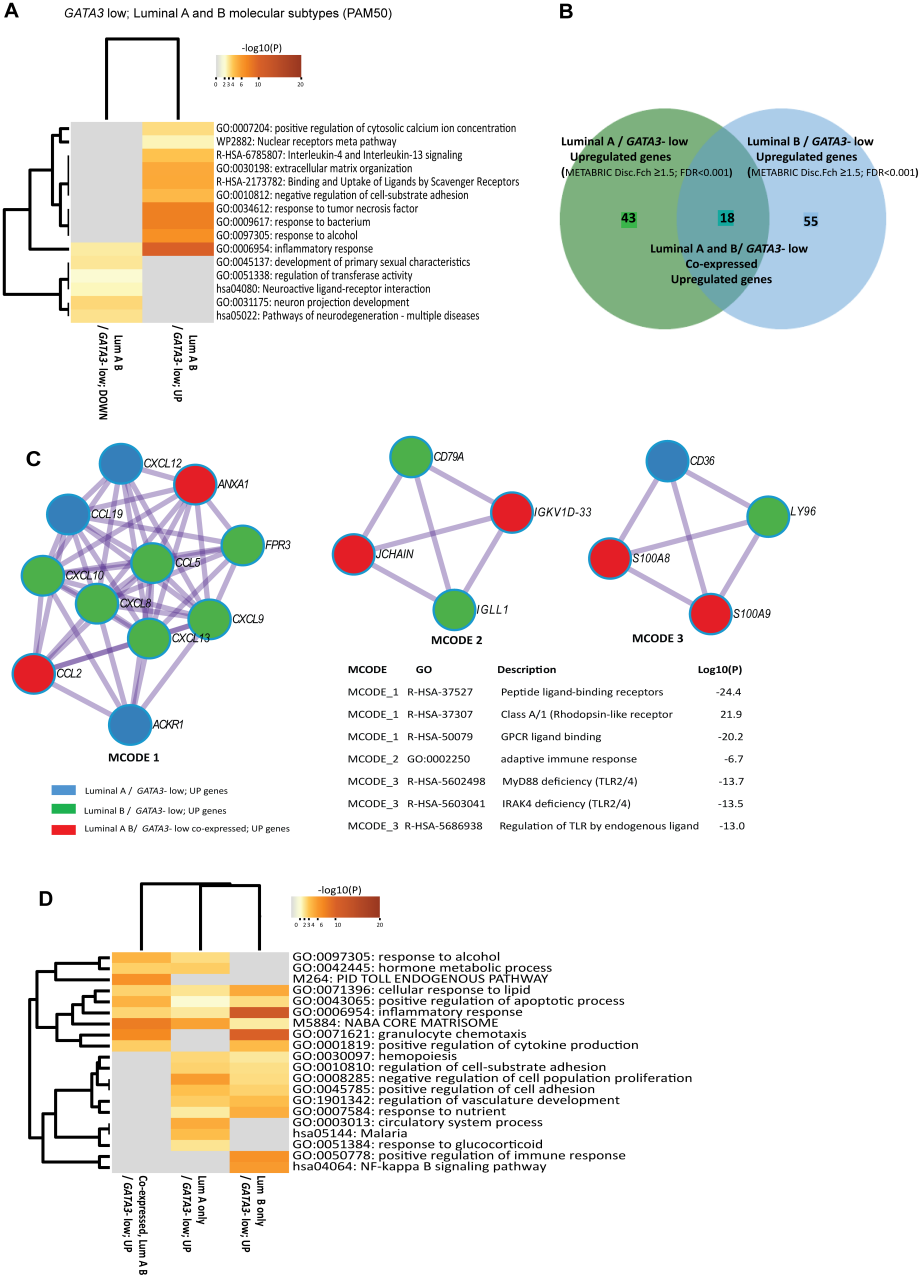
Pathway enrichment and molecular complex detection (MCODE) of protein–protein interaction (PPI) networks (by Metascape) across differentially expressed genes (DEGs) in *GATA3* high‐ and low‐cases in luminal molecular subtypes (PAM50). (A) Heatmap illustrating the top 20 clusters of functional pathway enrichment across up‐ and downregulated pathways in *GATA3*‐low, luminal A, and B combined. (B) Venn diagram depicting the number of DEGs upregulated in luminal A/*GATA3*‐low, luminal B/*GATA3*‐low, and co‐expressed genes in luminal A B/*GATA3*‐low. (C) Molecular complex detection (MCODE) analysis; GO enrichment illustrating densely connected PPI networks in association with *GATA3* low status in luminal A and B molecular subtypes (MCODE 1–3, genes color‐coded by associated luminal subgroup), and associated top ranked GO pathway enrichment of the components. (D) Heatmap of the top 20 clusters of functional pathway enrichment across *GATA3*‐low in luminal A, luminal B, and co‐expressed pathways. All heatmaps colored according to the *p* value. Input data was from Cohort II; METABRIC Discovery (*n*, number of patients = 734 (LumA and LumB); *n* = 466 (LumA); *n* = 268 (LumB); *GATA3* mRNA, two groups; cut‐off: lower quartile. SAM; Fold change ≥1.5; FDR <0.001).

Having explored *GATA3* expression in relation to the ER‐negative subgroup, we next investigated the role of GATA3 as a biomarker for immuno‐metabolic alterations in the luminal BC subtypes. Both immune‐ and metabolism‐associated genes were upregulated in *GATA3* low cases, and ‘Inflammatory response’ was the top‐ranked enriched pathway in *GATA3* low/luminal A and B combined (Figure 3A).

When comparing upregulated genes in *GATA3* low luminal B versus Luminal A samples, immune‐related genes (e.g., *GZMK*, *CD52*, complement marker *C1S*), in concert with genes reflecting metabolism (e.g., *APOD*, *FABP4*), are associated with the luminal A samples. Also, *CD36*, a fatty acid transporter gene implicated in tumorigenesis and immune modulation [51], was upregulated in luminal A tumors (Figure 3B; supplementary material, Table S7).

In luminal B tumors, immune‐related genes dominated among those upregulated in *GATA3*‐low cases, and markers of MDSCs (*S100A8/9*) were upregulated in both luminal A and B harboring low *GATA3* (supplementary material, Table S7). Densely connected protein–protein interaction (PPI) networks associated with chemokine signaling, adaptive immune responses, and immuno‐metabolic modulatory programs were top‐ranked pathway‐ and process‐enriched terms of the components in *GATA3* low luminal cases (Figure 3C).

When analyzing *GATA3*‐low associated genes and pathways in the luminal subtypes separately, the luminal B subtype demonstrated enrichment of ‘Granulocyte chemotaxis’ (Figure 3D; supplementary material, Figure S8), NF‐ĸB transcription factor targets (supplementary material, Figure S6A), gene regulators in the NF‐ĸB family, and *SPI1* was found in *GATA3*‐low/luminal B. *TWIST*2 and *GLI1*, described as associated with cancer stemness and EMT [52, 53], were the top ranked regulators in luminal A/*GATA3*‐low tumors (supplementary material, Figure S6B).

To validate the strong associations between GATA3 and immunological alterations, independent of ER status, we showed that loss of *GATA3* mRNA in both ER positive and negative tumors was associated with increased *CTLA4*, *PD1*, *PD‐L1*, *IDO1*, *LAG3*, *TIGIT*, and *TIM3* mRNA expression, and a high cytolytic activity (CYT) score (supplementary material, Table S8). In luminal A and luminal B molecular subtypes, low *GATA3* associated with increased expression of the immune checkpoint markers *CTLA4*, *PD1*, *IDO1*, the myeloid suppressor transcripts *S100A8/9*, and to high cytolytic activity by increased *GZMB* and CYT score (all *p* ≤ 0.008). *TIM3* expression associated inversely with *GATA3* expression in luminal B cases (*p* ≤ 0.001), and there was a significant but weaker association in luminal A tumors (*p* = 0.023). Of note, high *LAG3* associated with low *GATA3* in luminal B, but not in the luminal A molecular subtype. Low *GATA3* expression did not significantly associate with the expression of *PDL‐1* or *TIGIT* in the luminal subtypes (supplementary material, Figure S7).

DEGs and pathways reflecting adipogenesis and metabolic programs were associated with and enriched in *GATA3* low luminal tumors (Figure 3; supplementary material, Table S7 and Figure S3).

**Figure 4:**
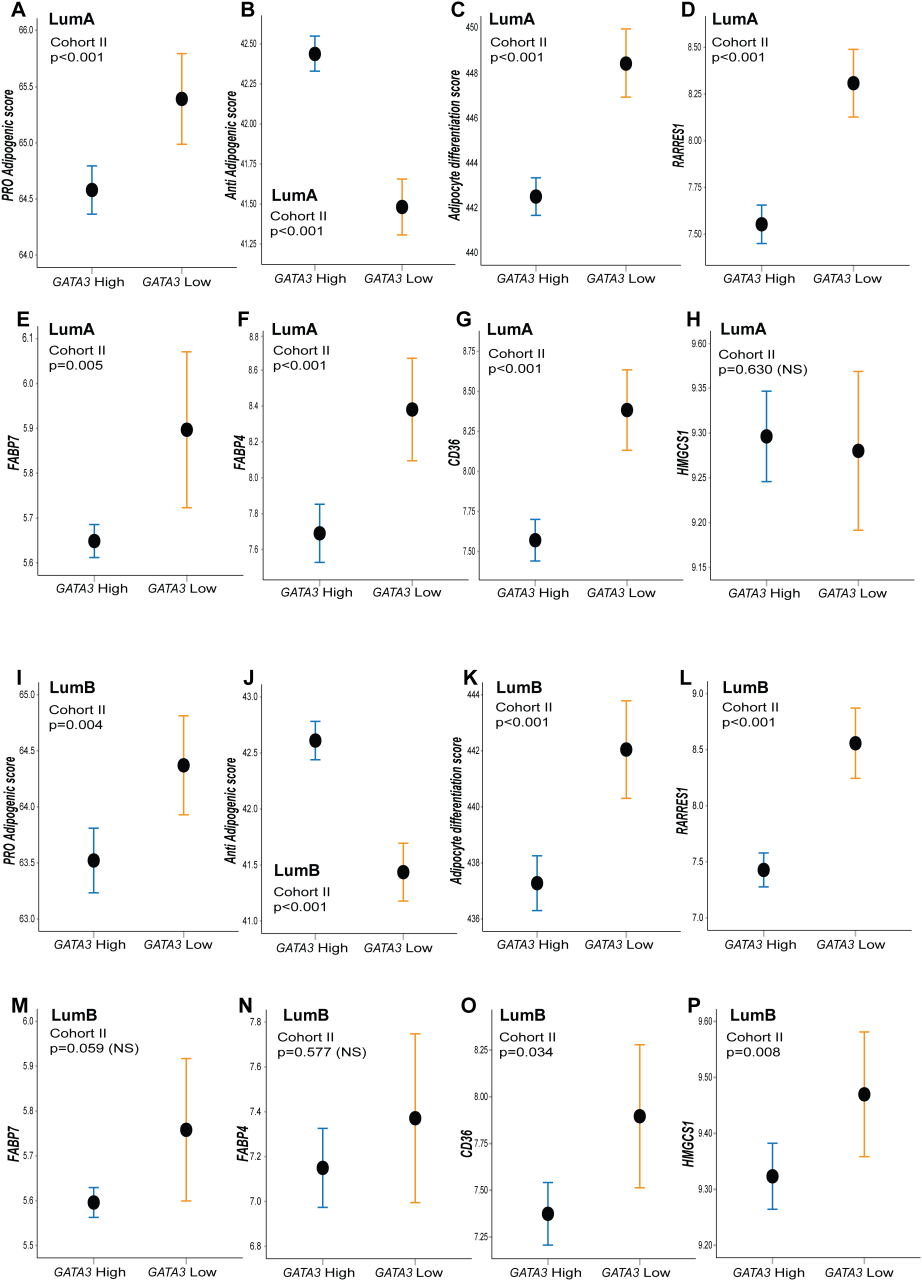
High and low *GATA3* mRNA expression in luminal molecular subtypes (PAM50); associations with biological programs reflecting adipogenesis and expression of selected metabolic transcripts. mRNA expression of a transcriptional pro‐adipogenesis score, an anti‐adipogenesis score, adipocyte differentiation score, and the metabolic transcripts *RARES1*, *FABP7*, *FABP4*, *CD36*, and *HMGCS1* across the expression of high (quartiles 2–4) and low (quartile 1) *GATA3* mRNA in (A–H) luminal A and (I–P) luminal B molecular subtypes. Error bars representing a 95% confidence interval from the mean, and *p* values by Mann–Whitney *U* test. All data from cohort II; METABRIC Discovery (*n*, number of patients; LumA, *n* = 466; LumB, *n* = 268. log2 transformed).

Further, we showed that low *GATA3* expression, independently in luminal A and luminal B molecular subtypes, was associated with a lower anti‐adipogenesis score and higher expression of the pro‐adipogenesis score and adipocyte differentiation score (all *p* < 0.001; Figure 4A–C, I–K). Members of the fatty acid binding protein family, *FABP7* and *FABP4*, showed increased expression in *GATA3* low/luminal A BC (*p* ≤ 0.005; Figure 4E, F, M, N, supplementary material, Table S7). The multifaceted lipid transporter *CD36* was upregulated in luminal A tumors and demonstrated increased expression in association with *GATA3* low luminal A cases (*p* < 0.001; Figure 4G). There was a weak association between low *GATA3* and increased *CD36* expression in the luminal B subgroup (*p* = 0.034). Of note, high *HMGCS1* was associated with low *GATA3* in luminal B (*p* = 0.008), but not in the luminal A molecular subtype (Figure 4H,P).

### Associations with drug‐induced signatures

Drug‐effect gene expression profiles were explored by querying the drug signature database Connectivity Map (CMAP; L1000) [43, 44], in breast cancer cell lines (MCF7 available only). Lovastatin and rosuvastatin were the top‐ranked compounds negatively enriched in *GATA3*‐low tumors (supplementary material, Table S9), indicating a potential relevance for HMGCR inhibitors to *GATA3*‐low BC. The farnesyltransferase inhibitor, Tipifarnib, was also among the top‐ranked compounds in the CMAP analysis (supplementary material, Table S9).

## Discussion

GATA3 expression is known to be associated with epithelial ER expression and luminal phenotypes in BC but is to a lesser extent examined in relation to other tumor programs, for example, processes in the TME. Here, we examined how GATA3‐low tumors are associated with features of immune responses and metabolic alterations, as well as basic tumor characteristics and clinical outcomes. As reported in previous studies [10, 11, 16, 18], we confirmed that low GATA3 protein and mRNA associate with aggressive BC and add novel findings on GATA3‐related immuno‐metabolic alterations not previously reported.

When elucidating the role of GATA3 independent of ER biology, we found that low *GATA3* was associated with a mixed immune response, with both stimulatory and inhibitory effects. As such, upregulation of several immune checkpoint genes was found (*CTLA4*, *ICOS*, *CD96*, and *CD27*), together with markers of cytolytic activity (*GZMA*, *B*, and *K*). In *GATA3*‐low tumors, independent of ER status, we observed an upregulation of genes reflecting non‐classical HLA, suggesting a role in immune suppression [54]. Increased expression of genes and interaction networks suggesting activation of the complement system was present [55], as well as interferon and chemokine signaling, indicating a composite immune response pattern in *GATA3*‐low tumors. Interestingly, in the ER‐negative as well as in the luminal B molecular subtype, members of the NF‐ĸB family were associated transcription factor targets for upregulated genes in *GATA3*‐low cases, independent of ER status. Taken together, our findings suggest an ER‐independent influence of GATA3 on immune responses in breast cancer. Also, we point to increased immunological alterations and a more active immunological profile in *GATA3*‐low luminal B versus luminal A tumors. To speculate, our findings could indicate a role for GATA3 in patient selection for targeted immunotherapy.

The relationship between immunological alterations and metabolic dysregulation has gained increased interest in cancer [56, 57]. It has been reported that adipocytes may induce pro‐inflammatory genes in triple negative BC cell lines [56], and elevated levels of PD‐L1 expression have been found in mature adipocytes [58]. To our knowledge, the potential role of GATA3 in cancer metabolism and BC‐associated adipogenesis has not previously been described. Here, we found inverse associations between *GATA3* gene expression and markers of lipid metabolism, signatures reflecting adipogenesis and adipocyte differentiation, independent of ER status. In support of this, obesity studies have suggested GATA3 as a negative transcriptional regulator of adipogenesis [11, 25, 27]. Interestingly, we found higher expressions of markers reflecting lipid uptake and transport (e.g., *FABP7*, *FABP4*, *CD36*) in *GATA3* low luminal A versus luminal B subtypes, suggesting differences in susceptibility to lipid accumulation. Potential immune modulatory interactions between CD36 and myeloid differentiation primary response protein (Myd88), interleukin associated kinase (IRAK), and Toll‐like receptor (TLR) signaling have been described [51], supporting that our results indicate the involvement of these programs in luminal tumors. As known, adipose tissue is a major site for cholesterol storage, and the mevalonate pathway is upregulated in several cancers, including BC [59].

In our study, we demonstrated an inverse association between *GATA3* and *HMGCS1*, a suggested marker of dysregulated mevalonate metabolism [50], in luminal B and ER‐negative tumors. Thus, the mevalonate pathway might be a druggable target, as described in several diseases, including cancers [60], and although the effects of statins are conflicting, improved survival has been observed for BC patients [60, 61]. Our *in silico* analyses identified several compounds with inhibitory effects on mevalonate metabolism (lovastatin, rosuvastatin, tipifarnib) that might be relevant in *GATA3*‐low BC. A recent study reported that Lovastatin inhibits EMT and metastasis in TNBC stem cells [62]. Further, Lovastatin has been reported to reduce lipid accumulation and adipocyte differentiation in mice [63], in support of a potential effect on reducing tumor‐associated adipogenesis in *GATA3*‐low tumors.

In addition to the cholesterol‐lowering properties of statins, studies are pointing to pleiotropic effects such as increased anti‐tumor immunity [64, 65], and a potential synergistic effect of statins in combination with immunotherapy is described in lung cancer [66]. Therefore, targeting the mevalonate pathway in GATA3‐low BC cases might have a dual role, as this could influence both metabolic alterations and the expression of immune checkpoint markers. Potential biomarkers of statin sensitivity in BC have been proposed [59, 67], and the relevance of GATA3 as a marker for response to statins in breast cancer treatment should be elaborated.

In addition to associations between *GATA3*‐low tumors and features of immune responses and metabolic alterations, we also confirmed known findings on tumor cell proliferation, stemness, EMT, and hypoxia in this BC subgroup [10, 11, 16, 18, 68]. Studies have demonstrated poorer survival in BC patients with tumors low in GATA3 protein and mRNA [20, 21], but have also reported no effect on survival when adjusting for ER status, indicating GATA3‐ER dependence [10, 19]. We found reduced BC‐specific and recurrence‐free survival in GATA3‐low cases (protein and mRNA), also among patients with luminal A tumors. In multivariate analysis, GATA3 protein and mRNA expression did not present independent prognostic value when adjusting for ER status in our data.

Our study is not without limitations. Notably, information regarding menopausal status and *BRCA* germline mutations was unavailable, factors which may potentially impact GATA3 function in BC. Furthermore, important confounding factors, like body mass index (BMI), physical activity, and treatment status are lacking, factors that could potentially interact with GATA3 function.

## Conclusion

We have shown that the loss of GATA3 is associated with aggressive tumor features in breast cancer, also independent of ER. To our knowledge, we present the first description of GATA3 in relation to tumor immuno‐metabolic alterations in BC. We suggest future studies to investigate GATA3 as a potential marker for response to immunotherapy and to evaluate the effects of statins, elucidating the relevance of adjuvant inhibition of the mevalonate pathway in GATA3‐low BC.

## Author contributions statement

EW conceived and designed the study. LAA and AKMS contributed to the study design. AKMS, EW, LAA and EAH contributed to data analysis, interpretation, and writing of the manuscript. AKMS, EW, IW and LAA contributed to immunohistochemical analysis and evaluation. KC, GK, AAS and CA performed the tissue‐based work and participated in data collection and interpretation. TA, AH, LMI ROCH and IMS contributed to data collection and interpretation. Collection, analyses, and interpretation of mRNA gene expression datasets were performed by EW and AKMS. Co‐authors gave critical input, and all authors read and approved the final submitted version of the paper.

## Supporting information


**Figure S1.** Flow chart summarizing the study cohorts and concept.
**Figure S2.** *GATA3* mRNA expression and correlations to ER‐related gene expression, expression of signatures reflecting hypoxia, and associations between *GATA3* expression, the *GATA3*‐low signature score, and molecular subtypes (PAM50). METABRIC Discovery cohort.
**Figure S3.** Recurrence free breast cancer survival by *GATA3* mRNA expression from the KM‐plotter database (www.kmplot.com).
**Figure S4.** Differentially expressed genes (DEGs) splitting *GATA3*‐low and ER‐negative tumors; *GATA3*‐low upregulated DEGs, associations to an immunogenic profile, independent of estrogen receptor. Molecular Complex Detection (MCODE) by Metascape. METABRIC Discovery cohort.
**Figure S5.** Associations between *GATA3* mRNA expression, selected metabolic transcripts and biological programs reflecting adipogenesis. METABRIC Discovery cohort.
**Figure S6.** Enrichment of transcription factor targets, and gene regulators across upregulated differentially expressed genes (DEGs) in *GATA3*‐low luminal A and luminal B molecular subtypes (PAM50). Analysis by Metascape; METABRIC Discovery cohort.
**Figure S7.** High and low *GATA3* mRNA expression in luminal molecular subtypes (PAM50) and associations to gene expression of immune checkpoint transcripts, markers for myeloid derived suppressor cells (MDSC), and cytolytic activity (METABRIC Discovery cohort).
**Figure S8.** Pathway enrichment across upregulated differentially expressed genes (DEGs) in *GATA3* high‐ and low cases in luminal molecular subtypes (PAM50), and differences between luminal A and luminal B tumors. Analysis by Metascape; METABRIC Discovery cohort.

**Table S1.** Associations between *GATA3* mRNA expression and selected characteristics in breast cancer. (A) METABRIC Discovery and (B) METABRIC Validation cohorts
**Table S2.** Significance analysis of microarray (SAM); up‐ and downregulated genes in *GATA3*‐low tumors, METABRIC Discovery cohort
**Table S3.** Significance analysis of microarray (SAM); *GATA3*‐low signature score, METABRIC Discovery cohort
**Table S4.** Top ranked gene sets enriched in *GATA3*‐low cases. Gene set enrichment analysis (GSEA)/molecular signature database (MSigDB)/C2 curated, METABRIC Discovery cohort
**Table S5.** Top ranked gene sets enriched in *GATA3*‐low cases. Gene set enrichment analysis (GSEA)/molecular signature database (MSigDB)/H, C2, C6, C5, C7, and KEGG C2 curated
**Table S6.** Genes upregulated in *GATA3*‐low tumors, not upregulated in ER‐negative (IHC) cases, METABRIC Discovery cohort; Venn analyses of differential expressed genes (DEGs)
**Table S7.** Genes upregulated in *GATA3*‐low luminal tumors, METABRIC Discovery cohort; Venn analyses of differential expressed genes (DEGs)
**Table S8.** Composite *GATA3* mRNA expression and ER status (by IHC). Associations with transcripts of immunological cytolytic activity (CYT) and immune checkpoint markers. METABRIC Discovery cohort
**Table S9.** Connectivity Map (L1000) analysis (METABRIC Discovery); Top 20 compounds with suggested effect on *GATA3*‐mRNA low tumors

## Data Availability

The public gene expression datasets (METABRIC cohorts) are available at https://ega-archive.org/studies/EGAS00000000083. Restrictions apply to new data generated within this study and are therefore not publicly available. However, upon reasonable request, interested researchers may contact the corresponding author to inquire about access. Request for non‐commercial use will be considered and will require full ethics review.
