# Supplementary material for: Reduced GATA3 expression associates with immuno‐metabolic alterations and aggressive features in breast cancer
Source: J Pathol Clin Res. 2025 Sep 29;11(6):e70050. doi: 10.1002/2056-4538.70050 (PMC12479720; doi:10.1002/2056-4538.70050)
Supplement: Supplementary file 1 — Figure S1. Flow chart summarizing the study cohorts and concept. Figure S2. GATA3 mRNA expression and correlations to ER‐related gene expression, expression of signatures reflecting hypoxia, and associations between GATA3 expression, the GATA3‐low signature score, and molecular subtypes (PAM50). METABRIC Discovery cohort. Figure S3. Recurrence free breast cancer survival by GATA3 mRNA expression from the KM‐plotter database (www.kmplot.com). Figure S4. Differentially expressed genes (DEGs) splitting GATA3‐low and ER‐negative tumors; GATA3‐low upregulated DEGs, associations to an immunogenic profile, independent of estrogen receptor. Molecular Complex Detection (MCODE) by Metascape. METABRIC Discovery cohort. Figure S5. Associations between GATA3 mRNA expression, selected metabolic transcripts and biological programs reflecting adipogenesis. METABRIC Discovery cohort. Figure S6. Enrichment of transcription factor targets, and gene regulators across upregulated differentially expressed genes (DEGs) in GATA3‐low luminal A and luminal B molecular subtypes (PAM50). Analysis by Metascape; METABRIC Discovery cohort. Figure S7. High and low GATA3 mRNA expression in luminal molecular subtypes (PAM50) and associations to gene expression of immune checkpoint transcripts, markers for myeloid derived suppressor cells (MDSC), and cytolytic activity (METABRIC Discovery cohort). Figure S8. Pathway enrichment across upregulated differentially expressed genes (DEGs) in GATA3 high‐ and low cases in luminal molecular subtypes (PAM50), and differences between luminal A and luminal B tumors. Analysis by Metascape; METABRIC Discovery cohort. [file CJP2-11-e70050-s001.pdf]

Reduced GATA3 expression associates with immuno-metabolic alterations and aggressive features in breast cancer

AKM Sæle *et al*. *J Pathol Clin Res* https://doi.org/10.1002/2056-4538.70050

Supplementary Figures S1–S8

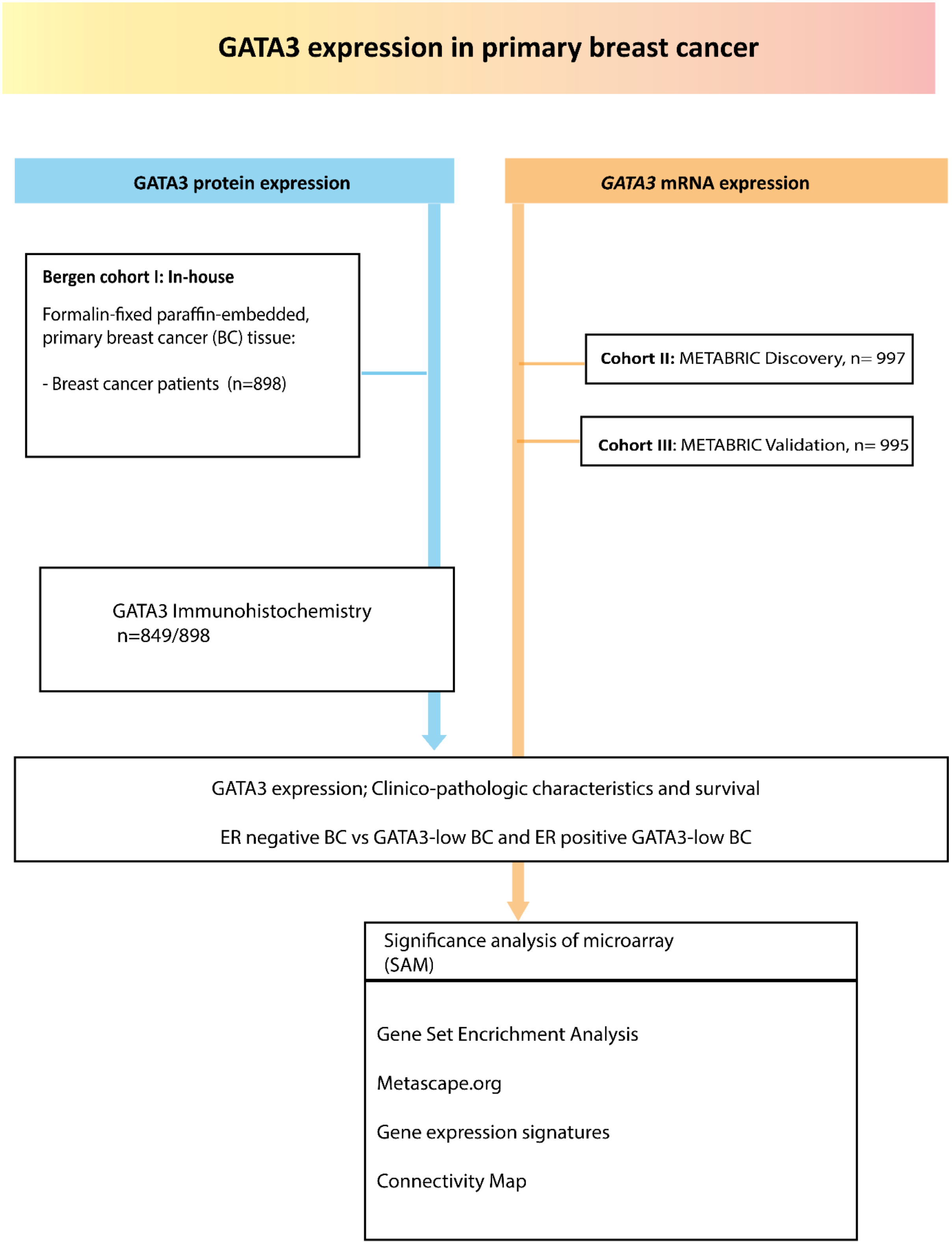

Figure S1. Flow chart summarizing the study cohorts and concept.

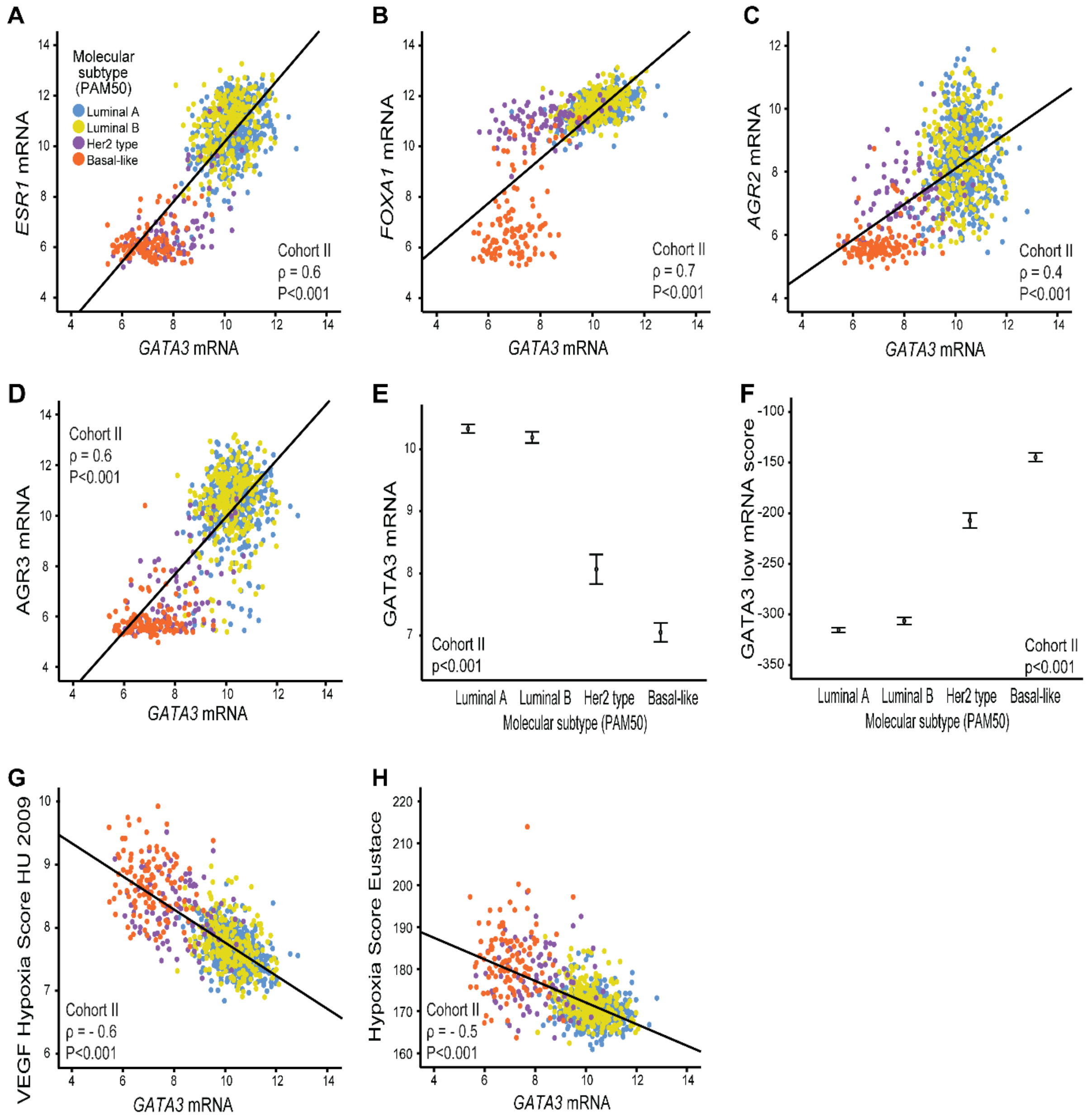

Figure S2. *GATA3* mRNA expression and correlations to ER-related gene expression, expression of signatures reflecting hypoxia, and associations between *GATA3* expression, the *GATA3*-low signature score and molecular subtypes (PAM50). Correlation between *GATA3* mRNA expression and (A) *ESR1*, (B) *FOXA1*, (C) *AGR2*, and (D) *AGR3*. (E, F) *GATA3* mRNA expression and *GATA3*-low mRNA signature score across PAM50 molecular subtypes. (G) *GATA3* mRNA expression and correlations to a VEGF hypoxia score and (H) a hypoxia signature score. Scatter plots with *p* values by Spearman's rank correlation and the coefficients (ρ). Error-bars represent 95% confidence intervals from the mean, and *p* values by Kruskal–Wallis test. All data from cohort II; METABRIC Discovery (*n*, number of patients = 939, log2 transformed). The normal breast-like category was excluded.

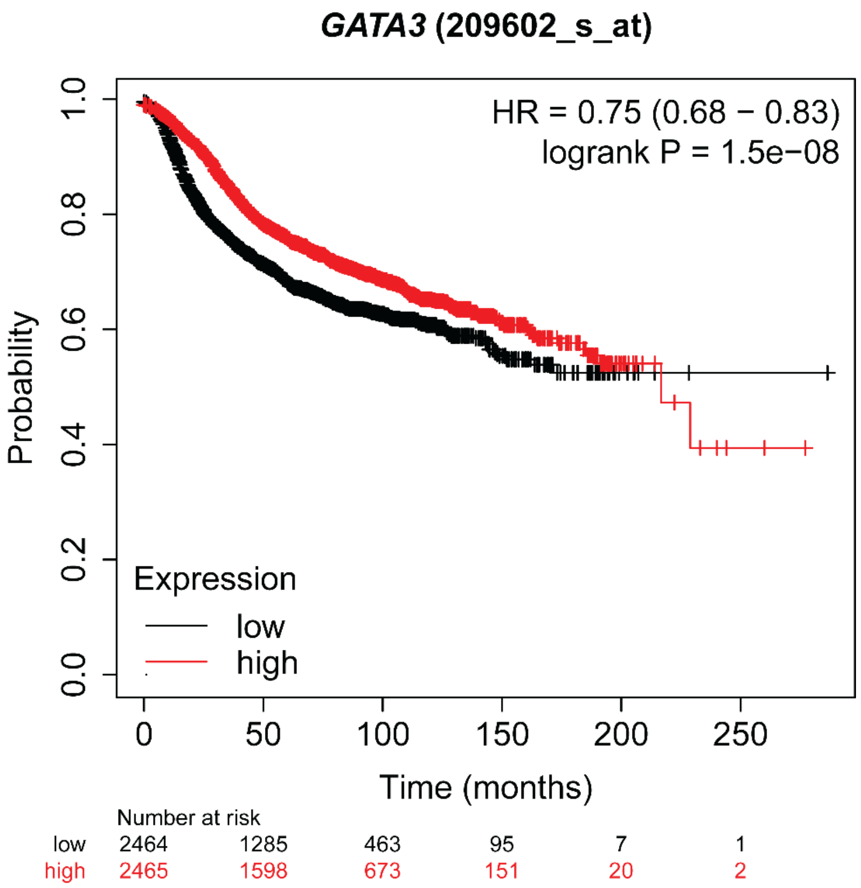

Figure S3. Recurrence free breast cancer survival by *GATA3* mRNA expression. Kaplan–Meier univariate survival according to *GATA3* mRNA expression in the cohorts from KM-plotter database (www.kmplot.com). Probe, *GATA3* (209602_s_at) – cut-off median, log-rank for test difference, end point; recurrence free survival (RFS).

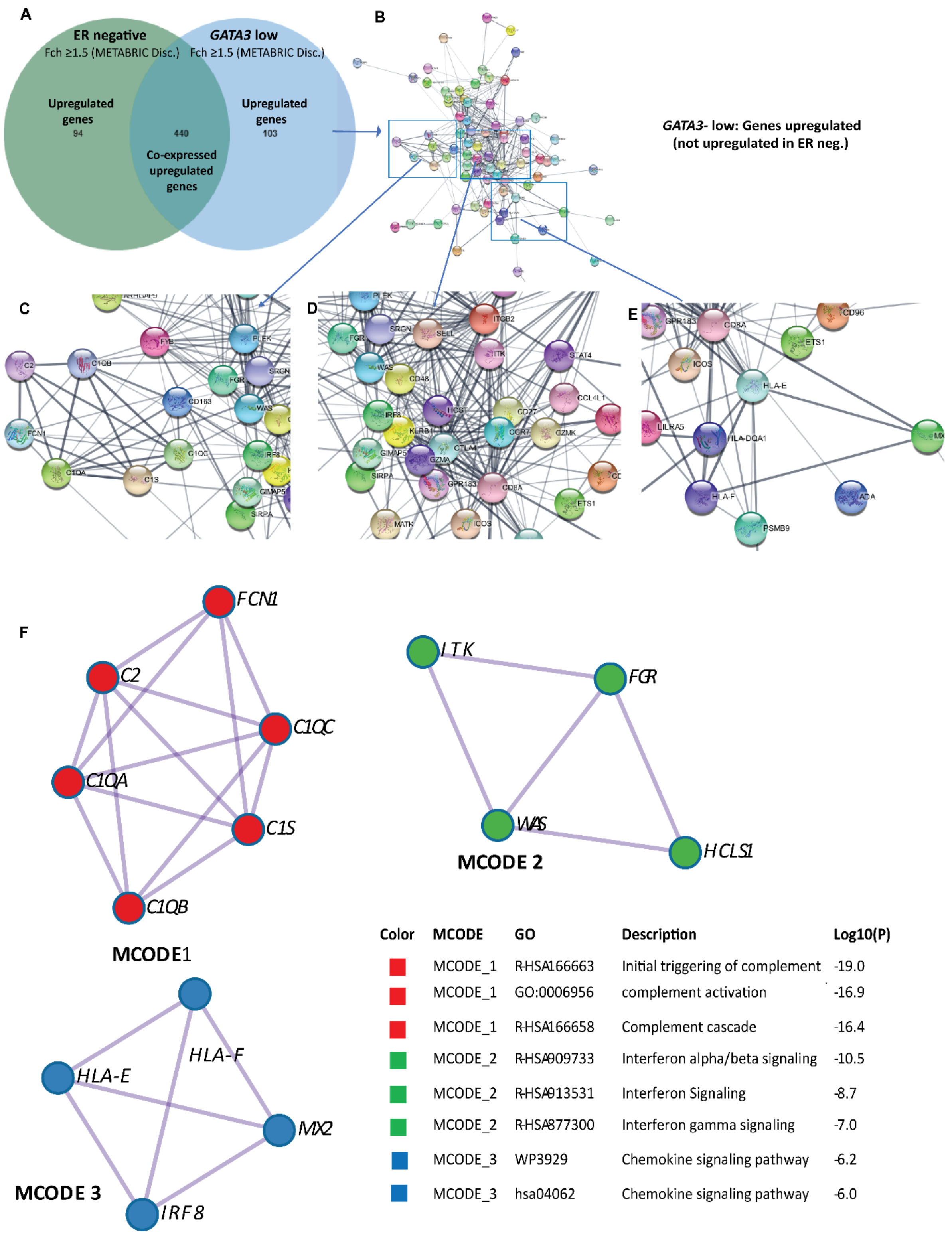

Color	MCODE	GO	Description	Log10(P)
	MCODE_1	R-HSA166663	Initial triggering of complement	-19.0
	MCODE_1	GO:0006956	complement activation	-16.9
	MCODE_1	R-HSA166658	Complement cascade	-16.4
	MCODE_2	R-HSA909733	Interferon alpha/beta signaling	-10.5
	MCODE_2	R-HSA913531	Interferon Signaling	-8.7
	MCODE_2	R-HSA877300	Interferon gamma signaling	-7.0
	MCODE_3	WP3929	Chemokine signaling pathway	-6.2
	MCODE_3	hsa04062	Chemokine signaling pathway	-6.0

Figure S4. Differential expressed genes (DEGs) splitting *GATA3*-low and ER-negative (IHC) tumors; *GATA3*-low upregulated DEGs, associations to an immunogenic profile, independent of estrogen receptor. Molecular Complex Detection (MCODE) by Metascape. (A) Venn diagram depicting number of DEGs upregulated in ER-negative and *GATA3*-low tumors, cut-off; Fold change ≥1.5, FDR <0.001. (B) Cytoscape string network illustrating genes upregulated in *GATA3*-low tumors, not co-expressed with ER-negatives (IHC), with (C) upregulation of genes reflecting compliment activation, (D) immune checkpoint molecules and cytolytic activity, and (E) HLA-related genes. MCODE

analysis; GO enrichment analysis, protein–protein network interactions illustrating densely connected network components enriched in association with *GATA3*-low status, not enriched in DEGs upregulated in ER negative cases. Data from Cohort II; Metabric Discovery (n = 937); SAM, Fold Change ≥1.5; FDR <0.001.

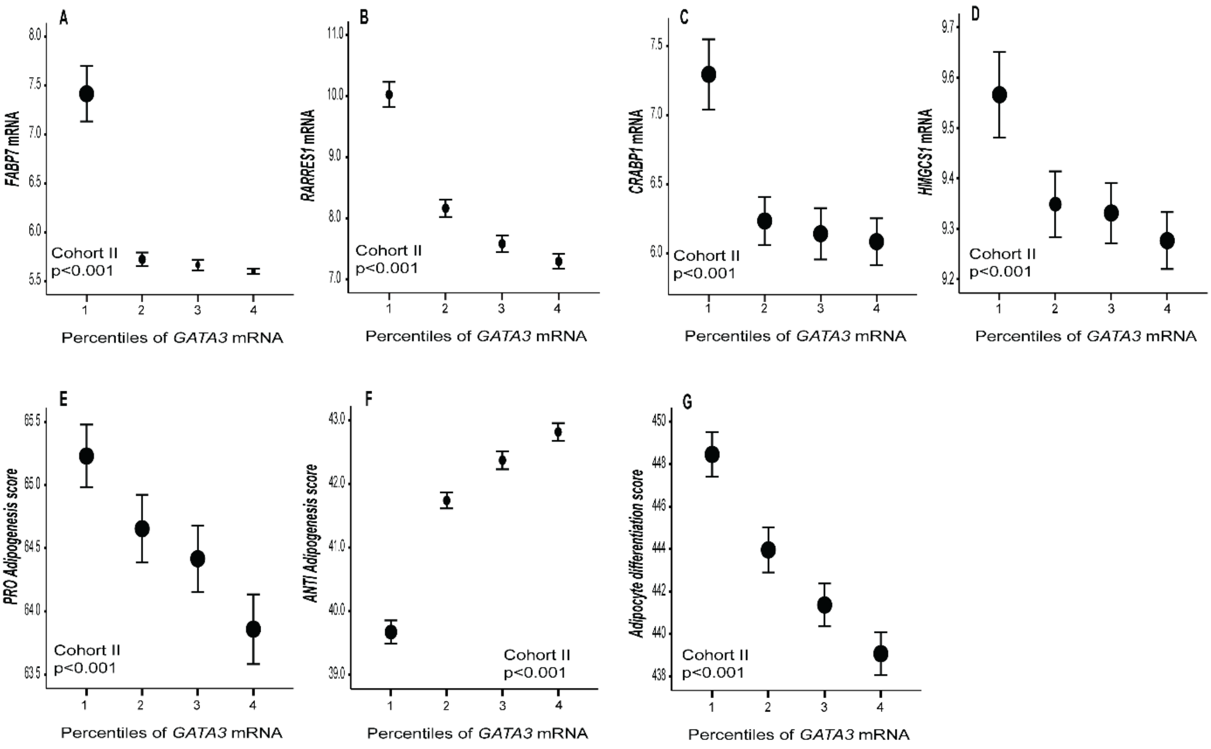

Figure S5. Associations between *GATA3* mRNA expression, selected metabolic transcripts and biological programs reflecting adipogenesis. mRNA expression of (A) *FABP7*, (B) *RARRES1*, (C) *CRABP1*, (D) *HMGCS1*, (E) a transcriptional pro-adipogenesis score, and (F) an anti-adipogenesis score derived from GSEA,MSigDB/C2/CP/WP_TRANSCRIPTIONAL_CASCADE_REGULATING_ADIPOGENESIS, and (G) an adipocyte differentiation signature score; URS_ADIPOCYTE_DIFFERENTIATION_UP across the expression of *GATA3* mRNA in percentile groups (quartiles). Error-bars representing 95% confidence interval from the mean, and *p* values by Kruskal–Wallis test. All data from cohort II; METABRIC Discovery (*n*, number of patients = 939, log2 transformed). The normal breast-like category was excluded.

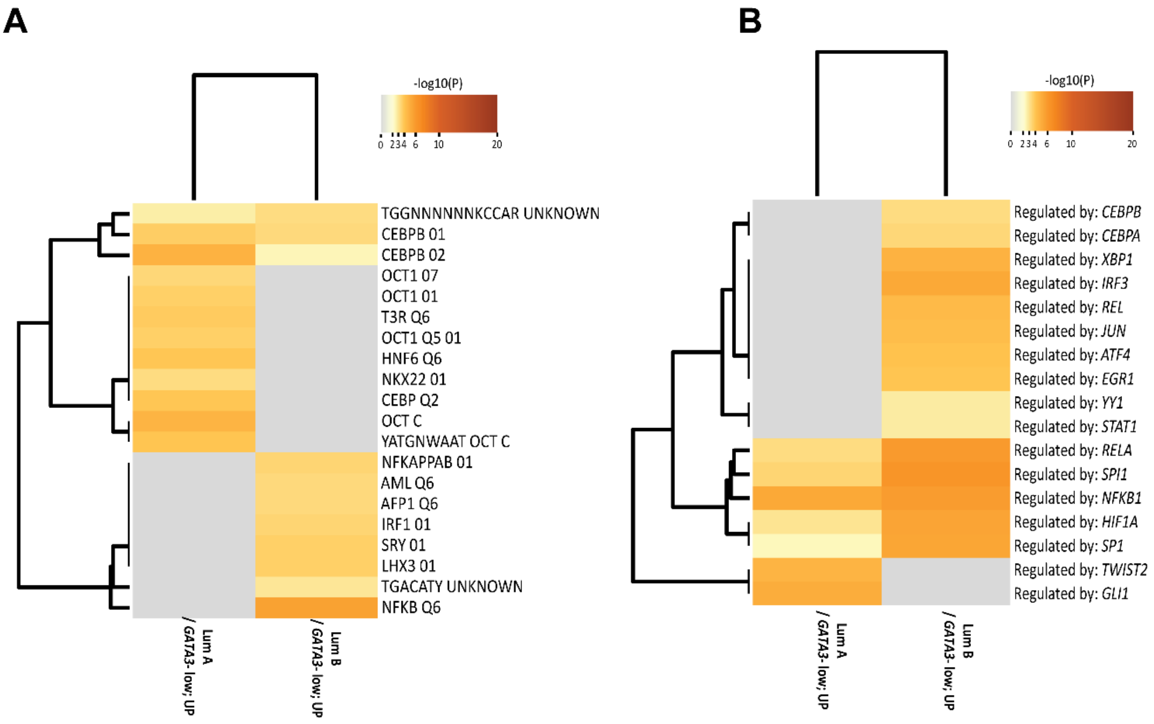

Figure S6. Analysis by Metascape; enrichment of transcription factor targets, and gene regulators across upregulated differentially expressed genes (DEGs) in *GATA3*-low luminal A and luminal B molecular subtypes (PAM50). (A) Heatmap illustrating enrichment of transcription factor targets across upregulated DEGs in *GATA3*-low luminal A and Luminal B molecular subtypes, and (B) enrichment of gene regulators across upregulated DEGs in *GATA3*-low cases, splitting luminal A and luminal B. All heatmaps colored according to the p-value. Input data was from Cohort II; METABRIC Discovery (*n*, number of patients = 734 (LumA and LumB); *n* = 466 (LumA); *n* = 268 (LumB); GATA3 mRNA, two groups (high/low); cut-off: lower quartile. Fold change ≥1.5; FDR <0.001).

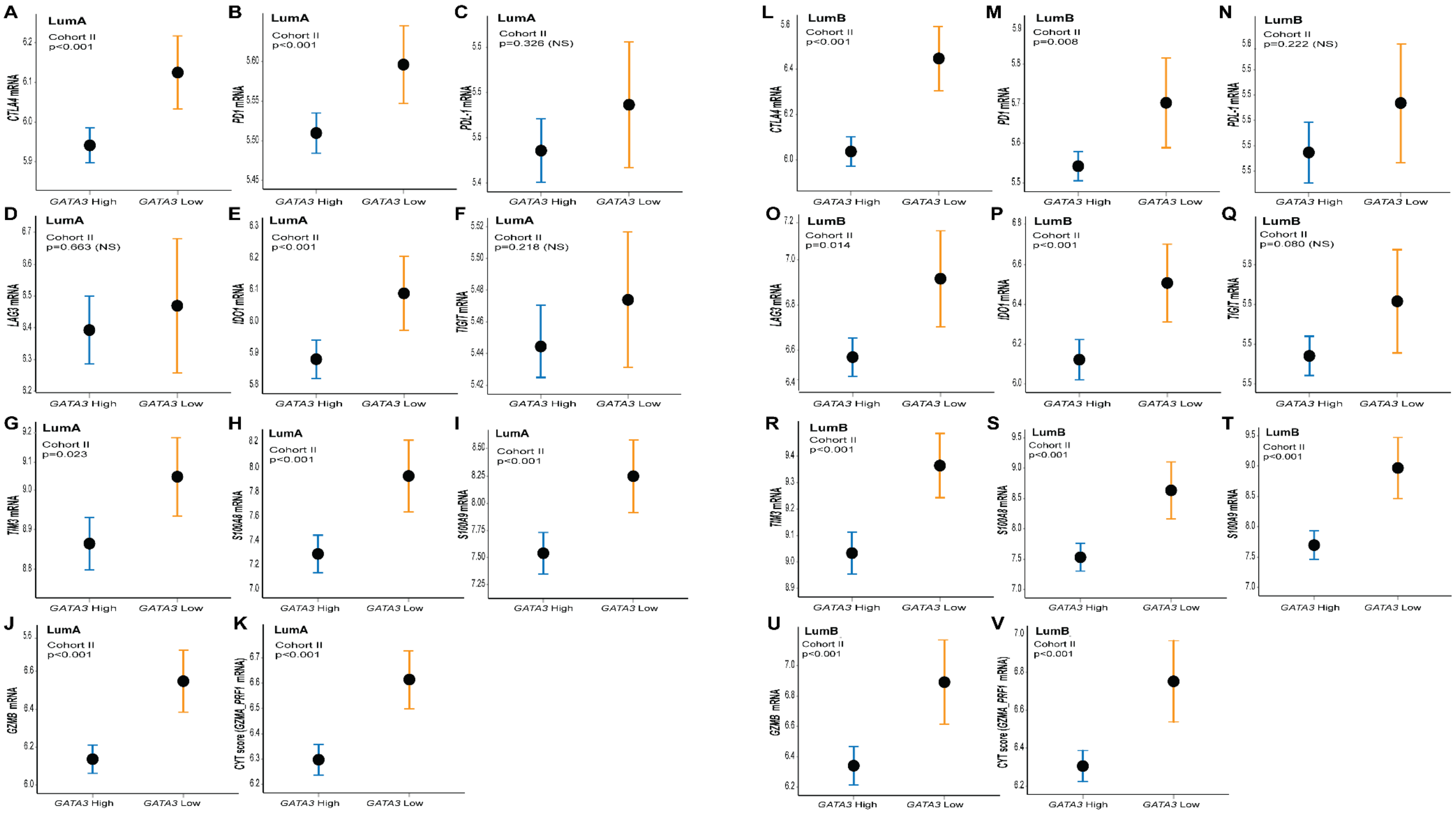

Figure S7. High and low *GATA3* mRNA expression in luminal molecular subtypes (PAM50) and associations with gene expression of immune checkpoint transcripts, markers for myeloid derived suppressor cells (MDSC), and cytolytic activity. mRNA expression of *CTLA4*, *PD1*, *PDL-1*, *LAG3*, *IDO1*, *TIGIT*, *TIM3*, *S100A8*, *S100A9*, *GZMB*, and cytolytic activity (CYT) score across the expression of high (quartiles 2–4) and low (quartile 1) *GATA3* mRNA in (A–K) Luminal A molecular subtype and (L–V) luminal B molecular subtypes. Error bars represent 95% confidence intervals from the mean, and *p* values by Mann–Whitney *U* test. All data from cohort II; METABRIC Discovery (*n*, number of patients; LumA, *n* = 466; LumB, *n* = 268. log2 transformed).

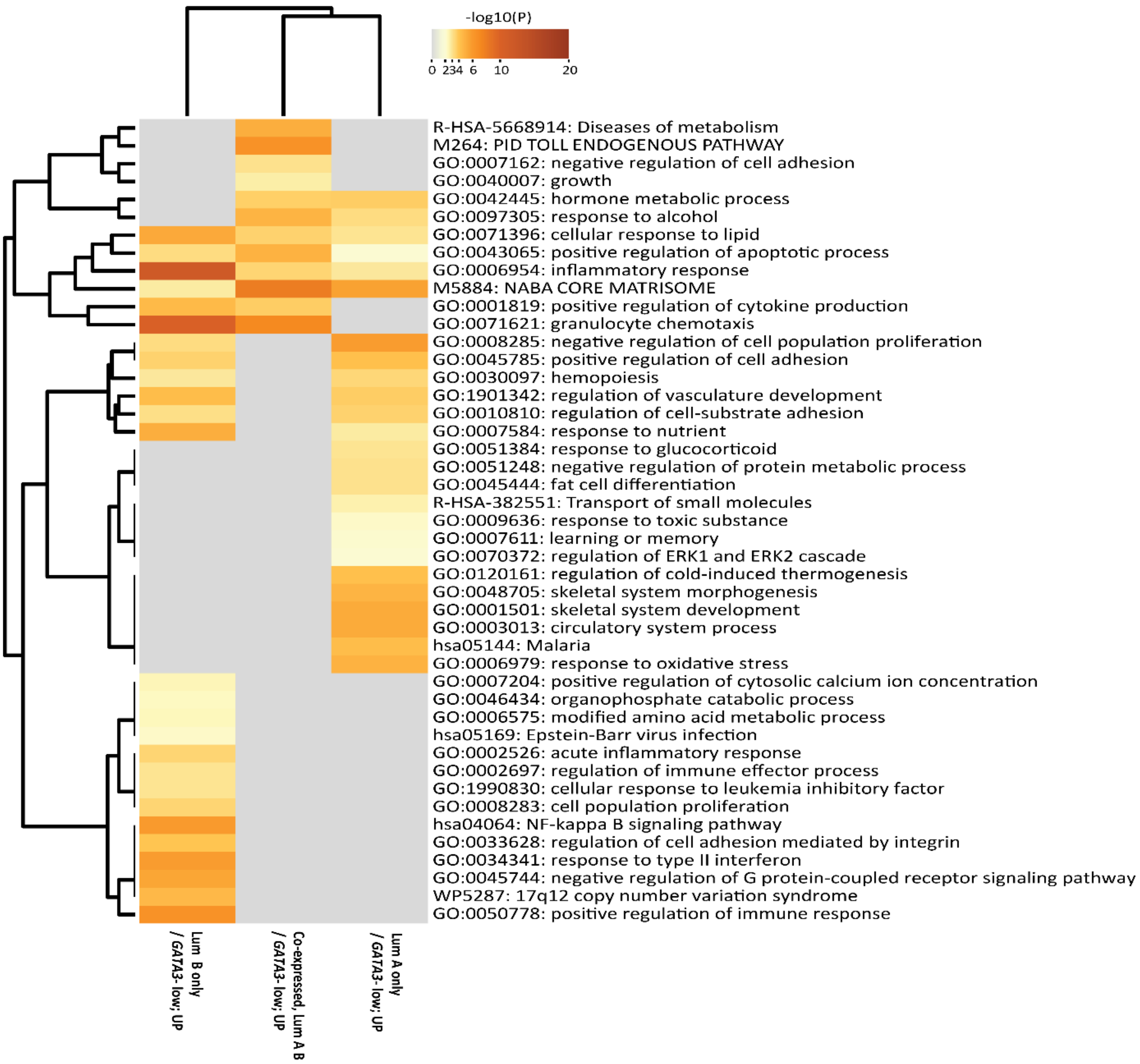

Figure S8. Analysis by Metascape; pathway enrichment across upregulated differentially expressed genes (DEGs) in *GATA3* high- and low cases in luminal molecular subtypes (PAM50), and differences between luminal A and luminal B tumors. Heatmap illustrating the top 100 clusters (extended list from Figure 3_Top20) of functional pathway enrichment across *GATA3*-low in luminal A, luminal B, and co- expressed pathways (luminal A and luminal B). All heatmaps colored according to the *p* value. Input data was from Cohort II; METABRIC Discovery [*n*, number of patients = 734 (LumA and LumB); *n* = 466 (LumA); *n* = 268 (LumB); *GATA3* mRNA, two groups; cut-off: lower quartile. SAM; Fold change ≥1.5; FDR <0.001].
