# Supplementary material for: Reduced GATA3 expression associates with immuno‐metabolic alterations and aggressive features in breast cancer
Source: J Pathol Clin Res. 2025 Sep 29;11(6):e70050. doi: 10.1002/2056-4538.70050 (PMC12479720; doi:10.1002/2056-4538.70050)
Supplement: Supplementary file 2 — Table S1. Associations between GATA3 mRNA expression and selected characteristics in breast cancer. (A) METABRIC Discovery and (B) METABRIC Validation cohorts Table S2. Significance analysis of microarray (SAM); up‐ and downregulated genes in GATA3‐low tumors, METABRIC Discovery cohort Table S3. Significance analysis of microarray (SAM); GATA3‐low signature score, METABRIC Discovery cohort Table S4. Top ranked gene sets enriched in GATA3‐low cases. Gene set enrichment analysis (GSEA)/molecular signature database (MSigDB)/C2 curated, METABRIC Discovery cohort Table S5. Top ranked gene sets enriched in GATA3‐low cases. Gene set enrichment analysis (GSEA)/molecular signature database (MSigDB)/H, C2, C6, C5, C7, and KEGG C2 curated Table S6. Genes upregulated in GATA3‐low tumors, not upregulated in ER‐negative (IHC) cases, METABRIC Discovery cohort; Venn analyses of differential expressed genes (DEGs) Table S7. Genes upregulated in GATA3‐low luminal tumors, METABRIC Discovery cohort; Venn analyses of differential expressed genes (DEGs) Table S8. Composite GATA3 mRNA expression and ER status (by IHC). Associations with transcripts of immunological cytolytic activity (CYT) and immune checkpoint markers. METABRIC Discovery cohort Table S9. Connectivity Map (L1000) analysis (METABRIC Discovery); Top 20 compounds with suggested effect on GATA3‐mRNA low tumors [file CJP2-11-e70050-s002.zip › cjp270050-sup-0002-TablesS1S4S5S8S9.pdf]

Reduced GATA3 expression associates with immuno-metabolic alterations and aggressive features in breast cancer

AKM Saele *et al*. *J Pathol Clin Res* https://doi.org/10.1002/2056-4538.70050

Supplementary Tables S1, S4, S5, S8, and S9

Table S1A. Cohort II (METABRIC Discovery): Associations between *GATA3* mRNA expression and selected characteristics in breast cancer (*n* = 939)

Variables	*GATA3* high (Q[a]2-4) *n* (%)	*GATA3* low (Q1) *n* (%)	OR	(95% CI)	*p* value [b]
**Age (≥/< 40 years)**					
≥40 years	682 (77.0)	204 (23.0)	1		
<40 years	23 (43.4)	30 (56.6)	4.36	(2.48 - 7.67)	<0.001
**Histological grade**					
Grade 1 and 2	424 (93.0)	32 (7.0)	1		
Grade 3	281 (58.2)	202 (41.8)	9.53	(6.37 - 14.24)	<0.001
**Tumor diameter**					
≤20 mm	317 (77.5)	92 (22.5)	1		
>20 mm	388 (73.2)	142 (26.8)	1.26	(0.93 - 1.70)	NS (0.148)
**Nodal status**					
Negative	385 (79.9)	97 (20.1)	1		
Positive	320 (70.0)	137 (30.0)	1.70	(1.26 - 2.29)	0.001
**ER**					
Positive (IHC)	688 (91.0)	68 (9.0)	1		
Negative (IHC)	17 (9.3)	166 (90.7)	98.80	(56.56 - 172.59)	<0.001
**PR**					
Positive (IHC)	473 (93.5)	33 (6.5)	1		
Negative (IHC)	232 (53.6)	201 (46.4)	12.42	(8.32 - 18.53)	<0.001
**Molecular subtypes (PAM50)**					
Lum A	441 (94.6)	25 (5.4)			<0.001
Lum B	249 (92.6)	19 (7.1)			
HER2 type	14 (16.1)	73 (83.9)			
Basal-like	1 (0.8)	117 (99.2)			

The normal breast-like category is excluded. [a] Q: quartiles; n: number of patients; OR: odds ratio; CI: confidence interval; [b]p- values by Pearson's chi-squared test; ER: estrogen receptor; PR; progesterone receptor. Missing information: none.

Table S1B. Cohort III (METABRIC Validation): Associations between *GATA3* mRNA expression and selected characteristics in breast cancer (*n* = 845)

Variables	*GATA3* high (Q[a]3-4) *n* (%)	*GATA3* low (Q1-2) *n* (%)	OR	(95% CI)	*p* value [b]
**Age (≥/< 40 years)**					
≥40 years	426 (53.9)	365 (46.1)	1		
<40 years	6 (11.1)	48 (88.9)	9.34	(3.95 - 22.07)	<0.001
**Histological grade**					
Grade 1. and 2.	264 (72.7)	99 (27.3)	1		
Grade 3.	125 (30.6)	284 (69.4)	6.05	(4.43 - 8.28)	<0.001
**Tumor diameter**					
≤20 mm	183 (52.7)	164 (47.3)	1		
>20 mm	249 (50)	249 (50)	1.11	(0.85 - 1.47)	NS (0.442)
**Nodal status**					
Negative	243 (55.2)	197 (44.8)	1		
Positive	189 (46.7)	216 (53.3)	1.41	(1.08 - 1.85)	0.008
**ER**					
Positive (IHC)	403 (68.8)	183 (31.2)	1		
Negative (IHC)	12 (5.4)	211 (94.6)	38.72	(21.10 - 71.08)	<0.001
**PR**					
Positive (IHC)	299 (70.2)	127 (29.8)	1		
Negative (IHC)	126 (30.9)	282 (69.1)	5.27	(3.92 - 7.08)	<0.001
**Molecular subtypes (PAM50)**					
Lum A	218 (85.5)	37 (14.5)			<0.001
Lum B	164 (73.2)	60 (26.8)			
HER2 type	43 (28.1)	110 (71.9)			
Basal-like	7 (3.3)	206 (96.7			

The normal breast-like category is excluded. [a] Q: quartiles; n: number of patients; OR: odds ratio; CI: confidence interval; [b]p- values by Pearson's chi-squared test; ER: estrogen receptor; PR: progesterone receptor. Missing information: Tumor grade: n=73; ER: n=36; PR: n=11.

Table S4. MSigDB [a]; Top ranked gene sets enriched in *GATA3*-low cases, (Cohort II; METABRIC Discovery)

Rank	Gene Set (GSEA/ MSigDB/ C2 curated)	Enrichment score	FDR [b] (%)
1	VANTVEER_BREAST_CANCER_ESR1_DN	0,9	0
2	YANG_BREAST_CANCER_ESR1_LASER_DN	0,86	0,05
3	BENPORATH_ES_CORE_NINE_CORRELATED	0,85	0
4	VANTVEER_BREAST_CANCER_METASTASIS_DN	0,84	0
5	COLLER_MYC_TARGETS_UP	0,84	0,05
6	SMID_BREAST_CANCER_RELAPSE_IN_BONE_DN	0,83	0
7	LIEN_BREAST_CARCINOMA_METAPLASTIC_VS_DUCTAL_UP	0,81	0
8	POOLA_INVASIVE_BREAST_CANCER_UP	0,79	0,01
9	BOSCO_TH1_CYTOTOXIC_MODULE	0,78	0,04
10	WORSCHECH_TUMOR_EVASION_AND_TOLEROGENICITY_UP	0,78	0,05
11	KEGG_AUTOIMMUNE_THYROID_DISEASE	0,78	0,05
12	LIM_MAMMARY_LUMINAL_PROGENITOR_UP	0,77	0
13	CHARAFE_BREAST_CANCER_LUMINAL_VS_BASAL_DN	0,76	0
14	REACTOME_CHEMOKINE_RECEPTORS_BIND_CHEMOKINES	0,76	0,05
15	FLORIO_NEOCORTEX_BASAL_RADIAL_GLIA_DN	0,75	0,05
16	RASHI_RESPONSE_TO_IONIZING_RADIATION_6	0,74	0
17	ALTEMEIER_RESPONSE_TO_LPS_WITH_MECHANICAL_VENTILATION	0,73	0,04
18	WANG_BARRETTS_ESOPHAGUS_AND_ESOPHAGUS_CANCER_DN	0,72	0,05
19	RUIZ_TNC_TARGETS_DN	0,71	0,03
20	REACTOME_SIGNALING_BY_SCF_KIT	0,71	0,05
21	PID_CD8_TCR_DOWNSTREAM_PATHWAY	0,71	0,05
22	KRIEG_HYPOXIA_VIA_KDM3A	0,7	0
23	CHARAFE_BREAST_CANCER_LUMINAL_VS_MESENCHYMAL_DN	0,7	0,03
24	SARRIO_EPITHELIAL_MESENCHYMAL_TRANSITION_UP	0,7	0,05
25	GHANDHI_BYSTANDER_IRRADIATION_UP	0,69	0
26	BASAKI_YBX1_TARGETS_UP	0,69	0
27	KEGG_NATURAL_KILLER_CELL_MEDIATED_CYTOTOXICITY	0,69	0
28	MARKEY_RB1_ACUTE_LOF_DN	0,69	0,03
29	BERTUCCI_MEDULLARY_VS_DUCTAL_BREAST_CANCER_UP	0,69	0,03
30	SHEDDEN_LUNG_CANCER_POOR_SURVIVAL_A6	0,69	0,05
31	FOURNIER_ACINAR_DEVELOPMENT_LATE_2	0,68	0
32	ZHU_CMV_ALL_UP	0,68	0,01
33	KEGG_CELL_ADHESION_MOLECULES_CAMS	0,68	0,03
34	ZHU_CMV_24_HR_UP	0,66	0,03
35	TARTE_PLASMA_CELL_VS_PLASMABLAST_DN	0,65	0,03
36	GINESTIER_BREAST_CANCER_20Q13_AMPLIFICATION_DN	0,65	0,05
37	GHANDHI_DIRECT_IRRADIATION_UP	0,64	0,03
38	GOLDRATH_ANTIGEN_RESPONSE	0,64	0,03
39	ZHANG_RESPONSE_TO_IKK_INHIBITOR_AND_TNF_UP	0,64	0,03
40	WANG_RESPONSE_TO_GSK3_INHIBITOR_SB216763_DN	0,63	0
41	RICKMAN_HEAD_AND_NECK_CANCER_E	0,63	0,03
42	HAHTOLA_SEZARY_SYNDROM_UP	0,63	0,05
43	BERENJENO_TRANSFORMED_BY_RHOA_UP	0,62	0,02
44	BREDEMEYER_RAG_SIGNALING_NOT_VIA_ATM_DN	0,62	0,03
45	RICKMAN_HEAD_AND_NECK_CANCER_C	0,62	0,04
46	KEGG_CYTOKINE_CYTOKINE_RECEPTOR_INTERACTION	0,61	0,03
47	FOSTER_TOLERANT_MACROPHAGE_UP	0,61	0,03
48	RICKMAN_METASTASIS_DN	0,61	0,03
49	ZHAN_MULTIPLE_MYELOMA_CD1_VS_CD2_UP	0,61	0,05
50	KEGG_TOLL_LIKE_RECEPTOR_SIGNALING_PATHWAY	0,61	0,05
51	WIERENGA_STAT5A_TARGETS_UP	0,6	0,05
52	BURTON_ADIPOGENESIS_2	0,6	0,05

Top ranked gene sets enriched in *GATA3*-low cases; C2 curated gene sets. GSEA: gene set enrichment analysis. [a] MSigDB: Molecular signature database; [b] FDR: false discovery rate (%).

Table S5. Top ranked gene sets enriched in *GATA3*-low cases; GSEA,MSigDB_H, C2, C6, C5, C7, and KEGG C2 curated. (Cohort II: METABRIC Discovery).

A)

Rank	Gene Set; GSEA/MSigDB/ C2 curated ALL	ES	FDR (%)
1	VANTVEER_BREAST_CANCER_ESR1_DN	0,9	0
2	YANG_BREAST_CANCER_ESR1_LASER_DN	0,86	0,05
3	BENPORATH_ES_CORE_NINE_CORRELATED	0,85	0
4	VANTVEER_BREAST_CANCER_METASTASIS_DN	0,84	0
5	COLLER_MYC_TARGETS_UP	0,84	0,05
6	SMID_BREAST_CANCER_RELAPSE_IN_BONE_DN	0,83	0
7	LIEN_BREAST_CARCINOMA_METAPLASTIC_VS_DUCTAL_UP	0,81	0
8	POOLA_INVASIVE_BREAST_CANCER_UP	0,79	0,01
9	BOSCO_TH1_CYTOTOXIC_MODULE	0,78	0,04
10	WORSCHECH_TUMOR_EVASION_AND_TOLEROGENICITY_UP	0,78	0,05
11	KEGG_AUTOIMMUNE_THYROID_DISEASE	0,78	0,05
12	LIM_MAMMARY_LUMINAL_PROGENITOR_UP	0,77	0
13	CHARAFE_BREAST_CANCER_LUMINAL_VS_BASAL_DN	0,76	0

B)

Rank	Gene Set; GSEA/ MSigDB, Hallmarks	ES	FDR (%)
1	HALLMARK_ALLOGRAFT_REJECTION	0,77	0
2	HALLMARK_INTERFERON_GAMMA_RESPONSE	0,75	0,33
3	HALLMARK_IL6_JAK_STAT3_SIGNALING	0,69	0,12
4	HALLMARK_INFLAMMATORY_RESPONSE	0,68	0,1
5	HALLMARK_E2F_TARGETS	0,68	0,45
6	HALLMARK_G2M_CHECKPOINT	0,66	0,23
7	HALLMARK_MTORC1_SIGNALING	0,61	0,19
8	HALLMARK_TNFA_SIGNALING_VIA_NFKB	0,61	0,36
9	HALLMARK_IL2_STAT5_SIGNALING	0,59	0,16
10	HALLMARK_COMPLEMENT	0,57	0,34
11	HALLMARK_KRAS_SIGNALING_UP	0,56	0,22
12	HALLMARK_MITOTIC_SPINDLE	0,55	0,17
13	HALLMARK_PI3K_AKT_MTOR_SIGNALING	0,53	0,2

C)

Rank	Gene Set; GSEA/MSigDB/ C5, GO BP	ES	FDR (%)
1	GO_GLUCOSAMINE_CONTAINING_COMPOUND_METABOLIC_PROCESS	0,83	0,03
2	GO_TOLL_LIKE_RECEPTOR_9_SIGNALING_PATHWAY	0,79	0,04
3	GO_NEGATIVE_REGULATION_OF_INNATE_IMMUNE_RESPONSE	0,78	0
4	GO_NATURAL_KILLER_CELL_MEDIATED_IMMUNITY	0,78	0,02
5	GO_REGULATION_OF_NATURAL_KILLER_CELL_MEDIATED_IMMUNITY	0,78	0,04
6	GO_REGULATION_OF_GRANULOCYTE_CHEMOTAXIS	0,75	0,05
7	GO_LYMPHOCYTE_COSTIMULATION	0,75	0,05
8	GO_MONOCYTE_CHEMOTAXIS	0,74	0,03
9	GO_REGULATION_OF_CELL_KILLING	0,73	0,03
10	GO_MONONUCLEAR_CELL_MIGRATION	0,71	0,03
11	GO_LEUKOCYTE_MEDIATED_CYTOTOXICITY	0,71	0,04
12	GO_RESPONSE_TO_INTERLEUKIN_12	0,71	0,04
13	GO_GRANULOCYTE_MIGRATION	0,7	0,05

D)

Rank	Gene Set; GSEA/MSigDB/ C7 immunological signature gene sets	ES	FDR (%)
1	GOLDRATH_EFF_VS_MEMORY_CD8_TCELL_UP	0,73	0,01
2	GSE1432_CTRL_VS_IFNG_24H_MICROGLIA_DN	0,73	0,01
3	GSE14000_UNSTIM_VS_4H_LPS_DC_TRANSLATED_RNA_DN	0,72	0
4	GSE7218_UNSTIM_VS_ANTIGEN_STIM_THROUGH_IGG_BCELL_DN	0,72	0,03
5	GSE15750_DAY6_VS_DAY10_EFF_CD8_TCELL_UP	0,72	0,05
6	GSE6259_FLT3L_INDUCED_DEC205_POS_DC_VS_CD8_TCELL_DN	0,71	0,01
7	GSE13547_CTRL_VS_ANTI_IGM_STIM_BCELL_2H_UP	0,71	0,02
8	GSE13484_UNSTIM_VS_YF17D_VACCINE_STIM_PBMC_DN	0,71	0,02
9	GSE18791_UNSTIM_VS_NEWCATSLE_VIRUS_DC_10H_DN	0,7	0,01
10	GSE37533_UNTREATED_VS_PIOGLIZATONE_TREATED_CD4_ TCELL_PPARG1_AND_FOXP3_TRASDUCED_UP	0,69	0
11	GSE21360_SECONDARY_VS_QUATERNARY_MEMORY_CD8_TCELL_UP	0,69	0
12	GSE37301_HEMATOPOIETIC_STEM_CELL_VS_CD4_TCELL_UP	0,69	0
13	GSE18281_CORTICAL_VS_MEDULLARY_THYMOCYTE_UP	0,69	0

E)

Rank	C2 curated KEGG enriched gene sets	ES	FDR (%)
1	KEGG_AUTOIMMUNE_THYROID_DISEASE	0,78	0
2	KEGG_NATURAL_KILLER_CELL_MEDIATED_CYTOTOXICITY	0,69	0
3	KEGG_CELL_ADHESION_MOLECULES_CAMS	0,67	0
4	KEGG_TOLL_LIKE_RECEPTOR_SIGNALING_PATHWAY	0,62	0
5	KEGG_CYTOKINE_CYTOKINE_RECEPTOR_INTERACTION	0,61	0
6	KEGG_FC_GAMMA_R_MEDIATED_PHAGOCYTOSIS	0,61	0
7	KEGG_LEUKOCYTE_TRANSENDOTHELIAL_MIGRATION	0,59	0,04
8	KEGG_B_CELL_RECEPTOR_SIGNALING_PATHWAY	0,63	0,05
9	KEGG_PRIMARY_IMMUNODEFICIENCY	0,86	0,06
10	KEGG_INTESTINAL_IMMUNE_NETWORK_FOR_IGA_PRODUCTION	0,8	0,06
11	KEGG_LEISHMANIA_INFECTION	0,72	0,06
12	KEGG_T_CELL_RECEPTOR_SIGNALING_PATHWAY	0,63	0,06
13	KEGG_CELL_CYCLE	0,6	0,06

F)

Rank	GSEA/MSigDB/ C6 oncogenic signature gene sets	ES	FDR (%)
1	HINATA_NFKB_IMMU_INF	0,81	0,1
2	CSR_LATE_UP.V1_UP	0,62	0
3	CORDENONSI_YAP_CONSERVED_SIGNATURE	0,62	0,09
4	SNF5_DN.V1_UP	0,61	0
5	KRAS.50_UP.V1_DN	0,61	0,05
6	RPS14_DN.V1_UP	0,61	0,06
7	RAF_UP.V1_UP	0,6	0
8	RPS14_DN.V1_DN	0,59	0
9	MTOR_UP.N4.V1_UP	0,58	0
10	BMI1_DN_MEL18_DN.V1_UP	0,58	0,05
11	ALK_DN.V1_UP	0,57	0
12	CSR_EARLY_UP.V1_UP	0,57	0,03
13	EGFR_UP.V1_UP	0,57	0,05

C2 curated gene sets ALL (**a**), H: Hallmarks (**b**), GO BP: Gene ontology biological process (**c**), C7 sub-collection immune Signature Database (**d**), C2 curated KEGG enriched gene sets (**e**), C6: Oncogenic signature gene sets (**f**). GSEA: Gene Set Enrichment Analysis; MSigDB: Molecular Signature Database; ES: enrichment score; FDR: false discovery rate.

Table S8. Cohort II (METABRIC Discovery): Composite *GATA3* mRNA expression and ER status (by IHC); Associations to transcripts of immunological cytolytic activity (CYT) score and immune checkpoint markers (*n* = 939)

Variables	ER pos / *GATA3* high [a] *n* (%)	ER pos / *GATA3* low [b] *n* (%)	ER neg / *GATA3* high *n* (%)	ER neg / *GATA3* low *n* (%)	*p* value [c]
**CYT score mRNA**					
Q 1	203 (29.5)	5 (7.4)	8 (47.1)	18 (10.8)	<0.001
Q 2	200 (29.1)	9 (13.2)	3 (17.6)	23 (13.9)	
Q 3	172 (25.0)	19 (27.9)	4 (23.5)	40 (24.1)	
Q 4	113 (16.4)	35 (51.5)	2 (11.8)	85 (51.2)	
***CTLA4* mRNA**					
Q 1	211 (30.7)	7 (10.3)	2 (11.8)	14 (8.4)	<0.001
Q 2	187 (27.2)	13 (19.1)	5 (29.4)	30 (18.1)	
Q 3	162 (23.5)	24 (35.3)	7 (41.2)	42 (25.3)	
Q 4	128 (18.6)	24 (35.3)	3 (17.6)	80 (48.2)	
***PD-L1* mRNA**					
Q 1	188 (27.3)	13 (19.1)	6 (35.3)	27 (16.3)	<0.001
Q 2	189 (27.5)	19 (27.9)	0 (0)	27 (16.3)	
Q 3	172 (25.0)	11 (16.2)	6 (35.3)	46 (27.7)	
Q 4	139 (20.2)	25 (36.8)	5 (29.4)	66 (39.8)	
***PD1* mRNA**					
Q 1	202 (29.4)	7 (10.3)	6 (35.3)	19 (11.4)	<0.001
Q 2	197 (28.6)	11 (16.2)	5 (29.4)	22 (13.3)	
Q 3	172 (25.0)	21 (30.9)	5 (29.4)	37 (22.3)	
Q 4	117 (17.0)	29 (42.6)	1 (5.9)	88 (53.0)	
***IDO1* mRNA**					
Q 1	218 (31.7)	5 (7.4)	3 (17.6)	8 (4.8)	<0.001
Q 2	208 (30.2)	10 (14.7)	6 (35.3)	11 (6.6)	
Q 3	168 (24.4)	17 (25.0)	6 (35.3)	44 (26.5)	
Q 4	94 (13.7)	26 (52.9)	2 (11.8)	103 (62.0)	
***LAG3* mRNA**					
Q 1	210 (30.5)	6 (8.8)	6 (35.3)	12 (7.2)	<0.001
Q2	196 (28.5)	17 (25.0)	2 (11.8)	20 (12.0)	
Q 3	170 (24.7)	18 (26.5)	6 (35.3)	41 (24.7)	
Q 4	112 (16.3)	27 (39.7)	3 (17.6)	93 (56.0)	
***TIGIT* mRNA**					
Q 1	195 (28.3)	11 (16.2)	6 (35.3)	22 (13.3)	<0.001
Q 2	192 (27.9)	13 (19.1)	4 (23.5)	26 (15.7)	
Q 3	188 (26.2)	16 (23.5)	3 (17.6)	36 (21.7)	
Q 4	121 (17.6)	28 (41.2)	4 (23.5)	82 (49.4)	
***TIM3* mRNA**					
Q 1	205 (29.8)	5 (7.4)	5 (29.4)	19 (11.4)	<0.001
Q 2	179 (26.0)	15 (22.1)	9 (52.9)	32 (19.3)	
Q 3	182 (26.5)	16 (23.5)	0 (0)	37 (22.3)	
Q 4	122 (17.7)	32 (47.1)	3 (17.6)	78 (47.0)	

The normal breast-like category is excluded. *n*: number of patients; [a-b] *GATA3*-low and -high: cut-off lower quartile; [c] *p* values by Pearson's chi-squared test; ER: estrogen receptor; Q: quartile; CYT-score: cytolytic activity score; *CTLA4*: cytotoxic T-lymphocyte associated protein 4; *PD-L1*: Programmed death-ligand 1; *PD1*: programmed death protein 1; *IDO1*: indolamine 2,3-dioxygenase 1; *LAG3*: lymphocyte activation gene 3; *TIGIT*: T-cell immunoreceptor with Ig and ITIM domains; *TIM3*: T-cell immunoglobulin and mucin-domain containing-3.

Table S9. Connectivity Map (L1000) analysis (METABRIC Discovery, Fch≥2); Top 20 compounds with suggested effect on *GATA3*- mRNA low tumors [a].

Rank	Enrichment score	CMAP Compound	Description
1	-99.92	Lovastatin	HMGCR inhibitor
2	-99.9	SC-9	Protein tyrosine kinase activator
3	-99.71	Rosuvastatin	HMGCR inhibitor
4	-99.67	PIK-90	PI3K inhibitor
5	-99.62	VAMA-37	DNA dependent protein kinase inhibitor
6	-99.61	WAY-213613	Glutamate inhibitor
7	-99.58	KU-0063794	MTOR inhibitor
8	-99.53	Tipifarnib	Farnesyltransferase inhibitor
9	-99.51	BRD-K64835161	-666 / CLK inhibitor
10	-99.45	Tetracycline	Bacterial 30S ribosomal subunit inhibitor
11	-99.43	Alisertib	Aurora kinase inhibitor
12	-99.34	DCEBIO	Potassium channel activator
13	-99.08	BJM-CSC-19	MEK inhibitor
14	-98.65	ZSTK-474	PI3K inhibitor
15	-98.6	PP-2	SRC inhibitor
16	-98.38	Reversine	Aurora kinase inhibitor
17	-98.38	OSI-027	MTOR inhibitor
18	-98.37	Wortmannin	PI3K inhibitor
19	-98.31	GDC-0941	PI3K inhibitor
20	-98.18	Urapidil	Adrenergic receptor antagonist

CMAP: connectivity map; Fch: fold change. [a] Compounds with gene-expression drug-effect profiles anticorrelated to low *GATA3* mRNA expression.
